# Mindfulness-Based Interventions for the Treatment of Substance and Behavioral Addictions: A Systematic Review

**DOI:** 10.3389/fpsyt.2018.00095

**Published:** 2018-03-29

**Authors:** Marta Sancho, Marta De Gracia, Rita C. Rodríguez, Núria Mallorquí-Bagué, Jéssica Sánchez-González, Joan Trujols, Isabel Sánchez, Susana Jiménez-Murcia, Jose M. Menchón

**Affiliations:** ^1^Department of Psychiatry, Bellvitge University Hospital-IDIBELL, Barcelona, Spain; ^2^Department of Psychiatry, Hospital de la Santa Creu i Sant Pau, Barcelona, Spain; ^3^Ciber Fisiopatologia Obesidad y Nutrición (CIBERobn), Instituto Salud Carlos III, Madrid, Spain; ^4^CIBER Salud Mental (CIBERSAM), Instituto de Salud Carlos III, Madrid, Spain; ^5^Department of Clinical Sciences, School of Medicine, University of Barcelona, Barcelona, Spain

**Keywords:** mindfulness, addiction, behavioral addiction, substance use, gambling

## Abstract

**Background:**

Emotion (dys)regulation as well as the interventions for improving these difficulties are receiving a growing attention in the literature. The aim of the present paper was to conduct a systematic review about the efficacy of mindfulness-based interventions (MBIs) in both substance and behavioral addictions (BAs).

**Method:**

A literature search was conducted using Cochrane, PubMed, and Web of Science. Fifty-four randomized controlled trials published in English since 2009 to April 2017 were included into a narrative synthesis.

**Results:**

Mindfulness-based interventions were applied in a wide range of addictions, including substance use disorders (from smoking to alcohol, among others) and BAs (namely, gambling disorder). These treatments were successful for reducing dependence, craving, and other addiction-related symptoms by also improving mood state and emotion dysregulation. The most commonly used MBI approaches were as follows: Mindfulness-Based Relapse Prevention, Mindfulness Training for Smokers, or Mindfulness-Oriented Recovery Enhancement, and the most frequent control group in the included studies was Treatment as Usual (TAU). The most effective approach was the combination of MBIs with TAU or other active treatments. However, there is a lack of studies showing the maintenance of the effect over time. Therefore, studies with longer follow-ups are needed.

**Conclusion:**

The revised literature shows support for the effectiveness of the MBIs. Future research should focus on longer follow-up assessments as well as on adolescence and young population, as they are a vulnerable population for developing problems associated with alcohol, drugs, or other addictions.

## Introduction

Nowadays, the incidence of behavioral addictions (BAs), such as gambling disorder, internet addiction, or compulsive buying, is increasingly important, even though substance use disorders (SUDs) are still the most prevalent addictions (1–6). BAs do not involve substance use but they share many core clinical features with substance addictions. In fact, in the latest version of the Diagnostic and Statistical Manual of Mental Disorders (7), gambling disorder appears in the “substance-related and addictive disorders” chapter, together with SUDs.

Lifetime prevalence rates of SUDs are estimated to be around 35.3% in the general population (8–10). When considering BAs these rates increase considerably (11). Moreover, addiction and other mental problems (especially mood and anxiety disorders) tend to often co-occur, maybe because the use of substances can actually induce mental disorders (12, 13) or because it can be a strategy of self-medication or simply an independent comorbid disorder. This situation generates an impairment in occupational and social functioning, as well as medical and legal problems. SUDs and BAs are characterized by the presence of a set of symptoms including tolerance, withdrawal, continued use despite wishes to stop, and despite knowing the negative consequences, a loss of regulatory control over drug cravings and further drug use [DSM-5 (7)]. Furthermore, *craving*, defined as “intense desire for drugs or addictive behaviors,” was added as diagnostic criteria and considered a key feature of emotion regulation that can affect drug use (10). Thus, SUDs are frequently associated with emotion regulation deficits and there seems to be a relationship between the severity of these deficits and higher drug use. However, the emotion dysregulation observed in addictions is not only described as an ongoing maintenance factor for drug use but also an early risk factor.

Following to Gross (14), “emotion regulation refers to the process of shaping the emotions that one has, when one has them, and how one experiences or expresses these emotions.” There are three core features of emotion regulation. First, there is an activation of a goal to modify the emotion-generative process (15, 16); second, it produces an engagement of the processes that are responsible for altering the emotion trajectory; and third, there is an impact on emotion dynamics (16, 17). Given the importance of this construct in addictions and in view of the current treatment limitations when approaching emotion (dys)regulation, other therapies have emerged for this aim including Mindfulness-based Relapse Prevention (18), Mindfulness Training (MT) for Smokers (MTS) (19), or Mindfulness-Oriented Recovery Enhancement [MORE (20)], and other therapies have been adapted to this population [e.g., Acceptance and Commitment Therapy, ACT (21)], Dialectical Behavior Therapy (22), or Mindfulness based Cognitive Therapy (23). MT represents the secular adaptation of Buddhist contemplative practices aimed to reduce suffering and foster well-being (24). Mindfulness has been described as “the awareness that arises from paying attention on purpose, in the present moment and non-judgementally to things as they are” [(25), p. 47] and unlike distraction, it is characterized by acceptance rather than withdrawal from aversive emotional experience (26).

The aim of this study is to conduct a systematic review of the efficacy of the mindfulness-based interventions (MBIs) in SUDs and BAs by focusing on randomized controlled trials. This review is necessary due to the inclusion of both behavioral and chemical addictions.

## Methods

This systematic review was conducted and reported in accordance with the Preferred Reporting Items for Systematic reviews and Meta-Analyses guidelines (27–29).

### Search Resources

Two independent reviewers conducted the literature search, including different sources such as electronic databases (Cochrane, PubMed, and Web of Science), citations, and reference lists, as well as gray literature. In addition, the reference lists of all included studies were hand searched, limiting the search to articles published in English. To ensure that articles were recent, the search was focused in trials published from 2009 to April 30, 2017.

The search terms used were a combination of MESH terms and keywords and included “mindfulness addiction,” “mindfulness based relapse prevention,” “mindfulness impulsivity,” “mindfulness substance abuse,” “mindfulness substance use,” “MBRP,” “mindfulness gambling disorder,” “mindfulness pathological gambling” in the title, abstract, or keywords.

### Eligibility Criteria

Eligibility criteria for the included studies were as follows: (1) to investigate the efficacy of MBIs in the samples of participants suffering from addictions, (2) inclusion of all ages, (3) to provide quantitative data supported by statistical methodology, (4) inclusion of a control group not receiving MBIs, (5) published in English, and (6) randomized controlled trials. Exclusion criteria were as follows: (1) quasi-experimental reports, (2) qualitative reports, (3) case reports, and (4) reviews (literature, systematic) and meta-analyses.

Mindfulness-based interventions were defined as a treatment addressed to promote the moment-by-moment awareness of thoughts, feelings, bodily sensations, and surrounding environment. It also involves acceptance, paying attention to thoughts and feelings without judging them.

### Study Selection

First, all included studies were screened based on their titles and abstracts by two reviewers. Second, the identified studies in this search were distributed between five reviewers. The extracted information was checked by one reviewer. The relevant studies were discussed in the case of a disagreement between reviewers.

### Data Items

The extracted information from each included trial was as follows: (1) characteristics of the participants (including gender, age, and diagnosis) and the inclusion and exclusion criteria; (2) type of intervention (including type, frequency, and duration; versus non-mindfulness intervention; or versus no treatment); (3) type of outcome measure (including validated scales for measuring and main related results); (4) length of follow-up; (5) dropout rates; and (6) limitations of these studies (see Appendix S1 and S2 in Supplementary Material).

Because of the variability between studies (e.g., heterogeneity of participants, interventions, and reported outcome measures), this systematic review focuses on describing these trials, their results, their applicability, and their limitations and on narrative synthesis rather than on conducting a meta-analysis.

### Outcome Measures

The primary outcome measure was the difference between MBIs and no MBIs on measures of reduction of addiction-related symptoms. Secondary outcome measures included changes in (1) self-reported mindfulness levels, (2) emotional self-regulation, (3) miscellaneous outcome measures, and (4) drop-out rates.

Findings are classified according to the specific addiction being explored in each study.

### Risk of Bias in Individual Studies

For substantiating the validity of the eligible randomized trials, two reviewers worked independently and reliably to determine the adequacy of randomization and concealment of allocation, blinding of patients, data collectors, loss to follow-up, and other sources of bias.

## Results

### Study Selection and Design

**Figure 1:**
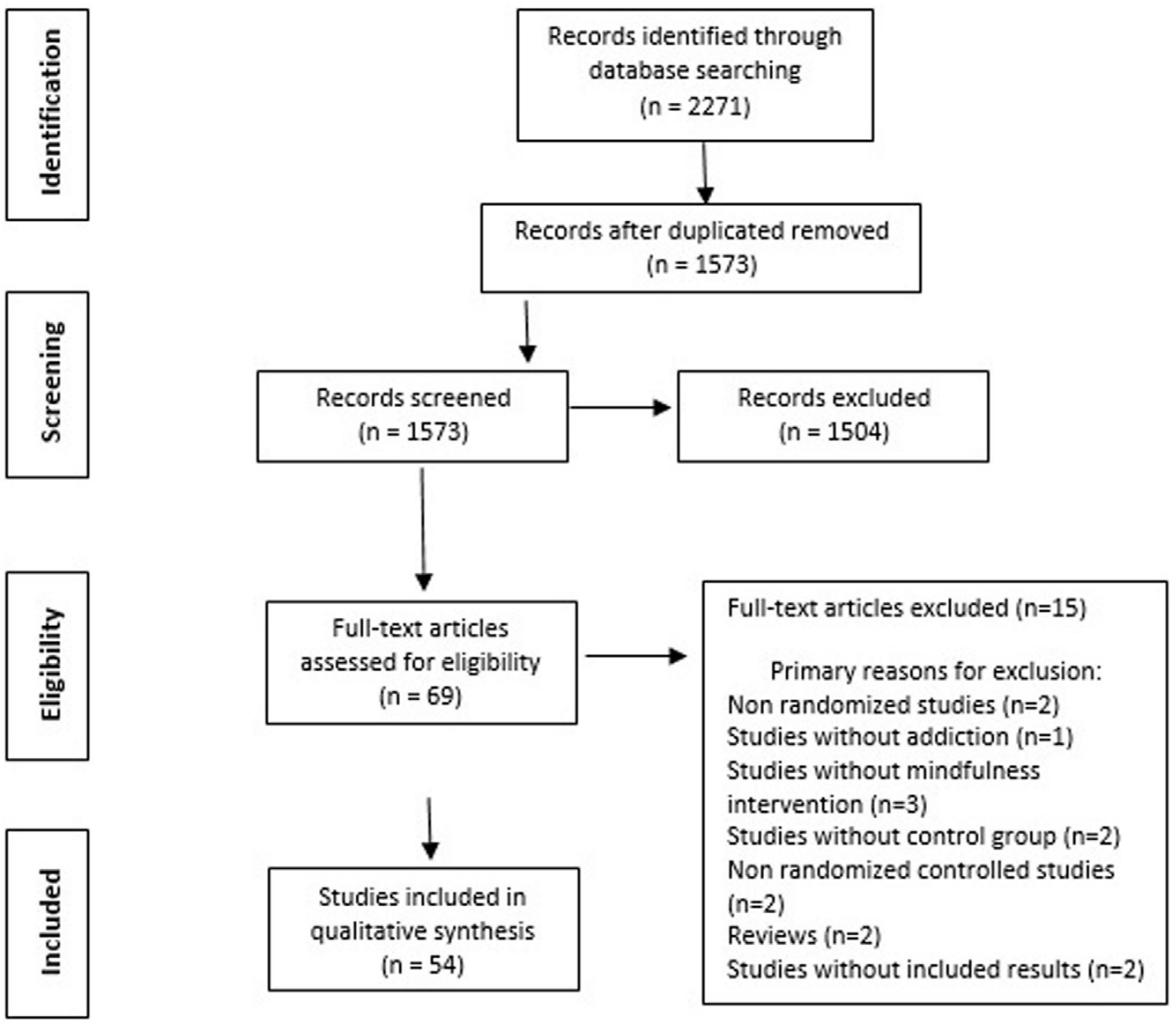
Preferred Reporting Items for Systematic reviews and Meta-Analyses flow chart of literature search.

The initial search identified 2,271 independent articles (see Figure 1 for the study selection flow chart). After removing duplicated and screened records, 69 full-text articles were assessed for eligibility and 15 of them were excluded for several reasons (e.g., no-randomized and/or controlled trials, reviews, no-mindfulness interventions, no addiction, or without included results). Finally, 54 articles met the criteria for inclusion and narrative synthesis (see Appendix S1 and S2 in Supplementary Material). All 54 studies finally selected for the review were randomized controlled trials published in English. The quality of these trials was evaluated.

Mindfulness interventions were applied in a wide range of addictions both in SUDs and BAs (e.g., gambling disorder). The majority of these studies focused on heterogeneous substance use, followed by studies on cigarette smoking, alcohol, opioids, gambling disorder, stimulants, marijuana, combination of cocaine and alcohol, and combination of tobacco and alcohol.

### Study Characteristics

#### Participants

The included studies involved 4,916 participants. The mean age of the participants was 34.95, and it was obtained from 51 out of the 54 studies because three trials did not provide age data (30–32). In 50 studies (out of 54), 57.89% were men and 42.11% were women. Four studies did not provide gender data (31, 33–35), five studies included only females (36–40) and six studies only males (41–46). Moreover, the target population of two studies was adolescence (47, 48) and of seven studies were young adults or college students (32, 34, 42, 44, 49–51). One trial (52) was focused on individuals with mild intellectual disabilities.

On the one hand, the most common inclusion criteria were (1) 18 years old or older; (2) English fluency; (3) meeting diagnostic criteria for SUD and other BAs (e.g., gambling disorder); (4) medical clearance; and (5) willingness to be randomized. However, several trials showed other or different inclusion criteria: (1) residency at the treatment center or therapeutic community (20, 30, 37); (2) to be able to speak and read Persian (53); (3) to be 18–29 (40, 50), 18–20 (42), 14 or older (48), 18–40 (33), 20–45 (41), and 21–29 years old (54); (4) living in low socioeconomic areas (55); (5) recurrent pain (56, 57); (6) adult with mild intellectual disability (52), and (7) having participated in a school-based intervention program (58). Furthermore, there were studies which did not provide these data (35, 44, 47, 59–63). On the other hand, the main exclusion criteria were (1) psychosis or other severe psychiatric disorders (e.g., bipolar disorder), (2) dementia, and (3) suicide risk. Nineteen trials did not provide the exclusion criteria (20, 30, 35–37, 42, 44, 46, 47, 49, 52, 54, 55, 60, 62, 64–67).

#### Intervention

The most frequent mindfulness interventions (Table 1) were MBRP (19, 36, 37, 45, 53, 60, 61, 68–72), MTS (50, 55, 73–75), MORE (20, 30, 43, 56, 57), ACT and variations (42, 62, 64, 66), and different types of Yoga (31, 47, 48).

**Table 1:** Relation of the main included mindfulness-based interventions (MBIs) in the search literature and the type of related addiction.

Main included MBIs	Studies	Type of related addiction
Mindfulness-Based Relapse Prevention	Bowen et al. (70)	Substance use disorder (SUD)
	Brewer et al. (19)	Cocaine and alcohol
	Carroll (71)	SUD
	Chawla et al. (60)	SUD
	Hsin Hsu et al. (61)	SUD
	Lee et al. (45)	SUD
	Glasner-Edwards et al. (72)	Stimulants
	Glasner et al. (76)	Stimulants
	Witkiewitz and Bowen (68)	SUD
	Witkiewitz et al. (36, 69)	SUD
	Witkiewitz et al. (37)	SUD
	Witkiewitz et al. (36, 69)	SUD
	Zemestani and Ottavia (53)	SUD

Mindfulness Training for Smokers	Brewer et al. (73)	Tobacco
	Davis et al. (50)	Tobacco and alcohol
	Davis et al. (55)	Tobacco
	Davis et al. (74)	Tobacco
	Kober et al. (75)	Tobacco

Mindfulness-Oriented Recovery Enhancement	Garland et al. (20)	Alcohol
	Garland et al. (30)	Alcohol
	Garland et al. (56)	Opioid
	Garland et al. (57)	Opioid
	Garland et al. (43)	SUD

Acceptance and Commitment Therapy	Bricker et al. (64)	Tobacco
	Dixon et al. (42)	Gambling disorder
	Luoma et al. (62)	SUD
	Smallwood et al. (66)	Opioid

Yoga	Butzer et al. (47)	SUD
	Butzer et al. (47)	SUD
	Fishbein et al. (48)	SUD
	Hallgren et al. (31)	Alcohol

Mindfulness-Based Stress Reduction	Reza and Hosseinalipour (46)	Opioid
	Vidrine et al. (77)	Tobacco

Mindfulness-Based Addiction Treatment	Vidrine et al. (78)	Tobacco

Mind-Body Bridging	Nakamura et al. (38)	SUD

Mindfulness-Based Group Therapy	Imani et al. (33)	Opioid

Integrative Body-Mind Training	Tang et al. (34)	Tobacco

Mindfulness Based Substance Abuse Treatment	Himelstein et al. (44)	SUD

Mindfulness-enhanced Cognitive Behavior Therapy	Toneatto et al. (79)	Gambling disorder

Dialectical Behavioral Therapy	Azizi et al. (41)	SUD

Affect Regulation Training	Stasiewicz et al. (80)	Alcohol

Mindful Awareness in Body-oriented Therapy	Price et al. (39)	SUD

Mindfulness Based Cognitive Therapy	Negrei et al. (35)	SUD

Non-specified mindfulness-based intervention	Alterman et al. (59)	SUD
	Vinci et al. (51)	Alcohol
	Bowen and Marlatt (49)	Tobacco
	Rogojanski et al. (65)	Tobacco
	Singh et al. (52)	Tobacco
	Murphy and MacKillop (54)	Alcohol
	Vernig and Orsillo (32)	Alcohol
	McIntosh et al. (63)	Gambling disorder
	Vinci et al. (67)	Alcohol
	Harris et al. (58)	Alcohol
	Valls-Serrano et al. (81)	SUD
	de Dios et al. (40)	Marijuana

Almost all interventions were composed of 7–12 weekly sessions with a duration of 1–3 h per session, were guided by two therapists, and were performed in a group format. Eight studies showed different characteristics. First, Bricker et al. (64) used a smartphone app-delivered ACT intervention for smoking cessation. Second, Fishbein et al. (48), Nakamura et al. (38), Butzer et al. (47), and Vinci et al. (51) implemented more intensive interventions (e.g., between 20 and 32 sessions). In third and last place, three trials conducted a single session intervention (32, 49, 54). In addition, two studies (52, 65) did not explain the characteristics of their interventions.

#### Comparator

The most frequently used control group in the included studies was Treatment as Usual (TAU) (31, 33, 38, 39, 44, 45, 48, 52, 53, 58–63, 68, 69, 77). Two studies (70, 71) compared MBRP with Relapse Prevention (RP) and TAU; and two studies more (36, 37) compared MBRP with RP. Cognitive Behavioral Treatment (19), Health Education [HE (66, 72, 76, 80)], Freedom from Smoking [FFS (73, 75)], Quit Line (74), Interactive Learning for Smokers [ILS (50)], Support Group [SG (20, 30, 56, 57)], a QuitGuide app (64), Physical Education (47), relaxation (34), medication treatment (naltrexone or others) (35, 41), and suppression (65) were other comparators. Several trials used two control groups: FFS and Quit Line Intervention (55), CBT and TAU (43, 78), relaxation and puzzle group (51, 67), distraction strategy and passive control (54), and TAU and healthy controls (32). Finally, six trials (40, 42, 46, 49, 79, 81) did not administer any treatment in the control groups (waiting list).

#### Outcomes

##### Primary

In the majority of studies, the primary outcome assessed was substance use (severity, abstinence, and craving). Chawla et al. (60) evaluated the adherence and competence of the intervention. Brain activation patterns and subjective ratings of slot machine outcomes during the fMRI tasks were the primary outcomes in the study by Dixon et al. (42), as well as in the studies by Toneatto et al. (79) and McIntosh et al. (63) gambling severity and urges were the primary outcomes. Smallwood et al. (66) assessed ACT in pain and addiction comorbidity using fMRI to evaluate neurophysiologic alterations across the treatment. In another study (46), the health-related quality of life (HRQOL) was the primary outcome. Finally, Negrei et al. (35) investigated if the combination of mindfulness techniques and a CBT group protocol diminished the level of depression and anxiety among a population with addictions.

##### Secondary and Additional Outcomes

These included levels of emotion regulation, dispositional mindfulness, distress tolerance, psychiatric severity, event-related brain potentials, general health, and mood. The timing of the outcome measures was variable and could include weekly assessment, post-treatment evaluation and 1, 2, 3, 4, 6, and 12-month follow-up evaluation. Sixteen studies did not report follow-up periods (20, 32, 33, 35, 38, 41–44, 46, 48, 57, 66, 67, 77, 81).

#### Attrition Rates

There was wide variability in the number of participants dropping out from MBIs with attrition rates ranging from 0 to 61% (median attrition = 23.765%). Some studies did not show attrition rates (31, 32, 35, 41, 42, 49, 54, 57, 60, 61, 63, 66, 67, 69, 72, 75).

## Discussion

Overall, the revised literature shows the value of MBIs for reducing dependence, craving, and other addiction-related symptoms as well as improving depression, anxiety, and perceived stress and emotion regulation difficulties. Nevertheless, in the majority of the included trials, the MBI effects do not persist at follow-up assessment. In addition, studies seem to indicate that a combination of a mindfulness intervention together with TAU (including active treatments) would be the best treatment option. With regard to the effects of MBIs, it has been observed that the combination of different processes such as acceptance, awareness, and non-judgment is necessary to predict changes in craving (69).

### Efficacy of MBIs Compared With TAU

In this review, several of the included studies compare MBIs with TAU. With regard to MBRP versus TAU, individuals who received MBRP and showed lower distress tolerance reported greater reductions in alcohol and other drugs use frequency over time (61) and craving scores (53, 69), although the changes were not maintained at the 4-month follow-up (61). Moreover, MBRP participants showed significantly higher negative expectancies of drug use and decreased depressive mood (45, 53) and anxiety (53) as well as no association between craving and depressive symptoms when compared to TAU individuals, who evidenced a strong association between these two lastly mentioned variables (68). Another result of these trials was a tendency toward a greater acceptance and less judgment when measured with Acceptance and Action Questionnaire (82) and Five-Facet Mindfulness Questionnaire (83) in MBRP participants (69).

For its part, Luoma et al. (62) examined the effects of ACT on shame when compared with TAU. They confirmed that the more gradual reductions in shame in the ACT group protected against high levels of substance use, and this intervention led to higher levels of outpatient treatment attendance during follow-up and continuous treatment gains, especially on psychosocial measures. The combination of other types of MBIs and TAU showed a significant reduction of urges to drink (31), and a greater degree of mindfulness was associated with less nicotine dependence and less withdrawal severity (52, 58, 77). Furthermore, the combination of MBIs and TAU provided a high self-efficacy regarding one’s ability to abstain from smoking in high-risk situations (77) and less craving and drug use in response to social pressure (39). MBIs reduced dissociation, perceived stress, and emotion regulation difficulties in a women population (39) and decreased medical problems over time in a meditation group although without long-term effects (59). Furthermore, the combination of MBIs and TAU decreased the primary outcome measure of drug and alcohol craving and the impact of past trauma and disturbed sleep as well as increased mindfulness, self-compassion and well-being (38), and self-esteem (44).

Finally, McIntosh et al. (63) explored the contribution of a MBIs + CBT for pathological gambling and whether the sequencing of these interventions impacted the effectiveness of the treatment (Mindfulness + CBT or CBT + Mindfulness). They found the combination of psychoeducation, mindfulness intervention, and CBT may be a useful complement of traditional CBT treatments, and it may be offered as an alternative treatment for gambling disorder to improve secondary dysfunction.

### Efficacy of MBIs Compared With CBT

While in treatment, participants in MBIs (e.g., MT) did not significantly differ from participants in CBT regarding treatment satisfaction, retention, or frequency of substance use, but MBI participants showed diminished physiological and psychological responses to stress provocation compared with the CBT group (19). In the study by Garland et al. (43), MORE intervention was associated with modest statistically significant improvements in post-traumatic stress symptoms, craving, and positive and negative affect from pre-to-post treatment versus the CBT group. Additionally, this intervention showed a significant indirect effect on craving and post-traumatic stress through increased dispositional mindfulness. In another study (78) that evaluated the efficacy of MBAT versus CBT and TAU, there were no significant overall differences in abstinence rates across the three treatments. However, MBAT showed benefits over CBT and TAU in promoting recovery from a lapse among participants who were not abstinent at the end of treatment. Still, Azizi et al. (41) observed that the cognitive therapy and naltrexone treatment were fewer effective than the emotion regulation treatment. Nevertheless, both mindfulness and cognitive therapy showed an increment in other symptoms such as distress tolerance or emotion regulation, and a decrement in the amount of drug abuse, anxiety, somatic symptoms, social dysfunction, and depression in comparison with pharmacotherapy. Furthermore, the combination of mindfulness and CBT (35) produced lower scores in depression and anxiety compared to the medication group.

### Efficacy of MBIs Compared With RP

One of the trials that compared MBIs with RP (70) assessed the relative efficacy of MBRP, RP, and TAU on 12-month SUD outcomes. MBRP and RP showed a decreased risk of relapse of drug and alcohol use. Compared with RP, the MBRP group showed an increased relapse risk of the first drug they used but RP and MBRP did not differ significantly on the time of the first heavy drinking. At the 3-month follow-up, no differences were found between groups; at the 6-month follow-up, RP and MBRP had a significantly reduced risk of drug use relapse and heavy drinking versus TAU, with an advantage in RP group over MBRP on the first drug use; and at the 12-month follow-up, MBRP reported significantly higher probability of not engaging in heavy drinking and fewer drug use days compared with RP. These results partially coincide with those of Witkiewitz et al. (36, 37), who found lower addiction severity and less drug use at follow-up in MBRP group versus RP. In the study by Carroll (71), MBRP was significant and positively related to mindfulness, whereas RP was significant and inversely related to mindfulness and associated with higher levels of thought suppression. Moreover, MBRP produced psychophysiological responses against stress that suggest self-regulation and self-soothing behaviors instead of abusing substances.

### Efficacy of MBIs Compared With HE

Mindfulness-Based Relapse Prevention and ART showed greater declines in negative affect (72, 76, 80), and MBRP is effective in reducing psychiatric severity (e.g., major depression and generalized anxiety disorder) as well as stimulant use among those with these health mental problems (72, 76). Stasiewicz et al. (80) found that the combination of CBT and ART demonstrated significantly greater increases of the percentage of abstinent days from the baseline to the tendency of treatment versus the combination CBT and HE even though this effect began to slightly decline during follow-up. Besides, MBIs (e.g., ACT) had neurophysiologic effects as the brain’s responsiveness to painful stimuli decreased in patients with chronic low back pain and opioid addiction comorbidity (66).

### Efficacy of MBIs Compared With Usual Smoking Interventions

There was variability with respect to the control groups of the included smoking studies. MBIs (e.g., MTS) showed significant improvements on self-reported measures of attentional control, emotion regulation, and mindfulness. Post-treatment, these measures were significantly correlated with meditation time and smoking abstinence versus Quit Line intervention or FFS (55, 73, 74). In addition, this type of mindfulness intervention (MTS) produced lower neural reactivity stress in regions including amygdala and insula (75).

When MTS was compared with ILS (50), there were no significant differences between groups in smoking abstinence although MTS participants showed significantly greater number of abstinence days in the first 2 weeks. With reference to alcohol use, controls significantly increased alcohol consumption over the course of the intervention, whereas MBI participants decreased consumption. For its part, in the study by Bricker et al. (64), a smartphone app-delivered ACT intervention for smoking cessation (SmartQuit) showed striking higher dropout rate versus a QuitGuide group control.

### Efficacy of MBIs Compared With SG

In a set of studies conducted by Garland et al. (20, 30, 56, 57), in which they compared MORE with a SG, they found stress and alcohol bias reduction and alcohol thought suppression as well as HRV recovery increase from alcohol cues following stress induction in MORE groups. Contrary to their hypotheses, MORE did not significantly increase self-reported mindfulness, nor did it result in significant decrements in craving, but significantly reduced symptoms associated with chronic pain and prescription opioid misuse.

### Efficacy of MBIs Compared With Non-Treatment

Dixon et al. (42) observed a neurological change in an ACT intervention toward similar brain activation patterns as non-pathological gamblers, including activation in the middle frontal gyrus and inferior parietal lobule. Following treatment, ACT participants were more likely to report higher engagement in psychological flexibility and mindfulness-related behaviors. In another study targeting problem gamblers (79), MBIs reported significantly fewer gambling symptoms, gambling urges, and psychiatric symptoms at post-treatment, and they demonstrated that the mindfulness practice (measured by number of minutes) was significantly correlated with a reduction in psychiatric symptoms. MBIs versus no treatment have been associated with negative affect and urges (49), improvements in working memory, reflection-impulsivity/decision-making and performance, reduced stress levels and increased planning (81), decreased marijuana use at post-treatment and 3-month follow-up in a women population (40), and better HRQOL due to increased awareness of thoughts and emotions, acceptance, and compassion (46).

### Efficacy of MBIs Compared With Other Interventions

Mindfulness-based interventions have been compared with numerous control interventions. With reference to yoga versus physical education, Butzer et al. (47) concluded that yoga has beneficial effects on emotional self-control (females) and willingness to smoke cigarettes (females and males). No differences between groups pre- to post-interventions were found although they observed improvements over an extended period of time.

In college students, MBIs compared with relaxation (34, 51, 67) showed a significant smoking and craving reduction, an increased activity at inferior frontal gyrus/ventrolateral PFC and ACC/medial PFC and a decreased activity at cerebellum, posterior cingulated cortex/precuneus, and other regions after the intervention. Furthermore, MBIs were effective at increasing mindfulness state, and MBIs such as relaxation showed decreased negative affect and urge after interventions. However, higher levels of some of the facets of impulsivity, such as Sensation seeking, Negative and Positive urgency were associated with increased negative affect and urge and decreased positive affect in the post-mindfulness intervention.

When MBIs were compared to a distraction strategy or passive control or suppression condition (54, 65), there were no overall differences between groups. It seems that dispositional anxiety sensitivity did not have an impact on the outcomes but, state symptom-focused anxiety immediately after the cue induction procedure was a significant predictor of self-efficacy at follow-up. Specifically, and contrary to previous research, individuals who are anxiety sensitive do equally well, or better, when coping with cravings using a suppression-based approach as they do when using a mindfulness strategy, at least in the short term.

## Limitations

The systematic review reported here intent to show MBIs’ results and effects. The main limitation of this review is that the patient population, MBIs, comparators, and outcome measures are not the same across studies. Moreover, the quality was adequate in all studies. Another limitation of our study is the number of trials included in the review, 54, and the consequent difficulty to integrate all information. However, for solving this problem we used five reviewers.

## Conclusion and Future Perspectives

The revised literature gives support to the effectiveness of the MBIs. These treatments are adequate to reduce dependence, craving, and other addiction-related symptoms as well as to improve mood state and emotion dysregulation. There are certain interventions that presented better results in the treatment of addiction such as MBRP, MTS, or MORE. Nevertheless, the best effectiveness may be the combination of the MBIs with TAU or another active treatment. Besides, few studies have found maintenance of the effects over time, and it would be important to conduct more follow-up studies. As for the target population, it would be interesting to investigate addiction problems in adolescents and young adults because they are a fragile and sensitive population to develop special interests in drugs and other addictions.

## Author Contributions

Research project elaboration: MS, SJ-M, IS, and JM; organization: MS; execution: MS, MG, CR, NM-B, and JS-G; design: MS and CR; writing of the first draft: MS; review and critique: MS, MG, CR, NM-B, JS-G, SJ-M, IS, and JT.

## Conflict of Interest Statement

The authors declare that the research was conducted in the absence of any commercial or financial relationships that could be construed as a potential conflict of interest.
