# Supplementary material for: Mindfulness-Based Interventions for the Treatment of Substance and Behavioral Addictions: A Systematic Review
Source: Front Psychiatry. 2018 Mar 29;9:95. doi: 10.3389/fpsyt.2018.00095 (PMC5884944; doi:10.3389/fpsyt.2018.00095)
Supplement: Supplementary file 1 [file data_sheet_1.doc]

Supplementary Material

**Mindfulness-based interventions for the treatment of substance and behavioral addictions. A systematic review.**

Sancho M.*^1,2^*, De Gracia M.*^1^*, Rodríguez R.C.*^1^*, Mallorquí-Bagué N.*^1,3^*, Sánchez-González J.*^1^*, Trujols J. *^2,4^*, Sánchez I. *^1^*, Jiménez-Murcia S. *^1,3,5^,* Menchón J.M.*^1,4,5^*

*1 Department of Psychiatry. Bellvitge University Hospital-IDIBELL, Barcelona, Spain*

*2 Department of Psychiatry, Hospital de la Santa Creu i Sant Pau, Barcelona, Spain*

*3 CIBER Fisiopatologia Obesidad y Nutrición (CIBERobn), Instituto Salud Carlos III, Madrid, Spain*

*4 CIBER Salud Mental (CIBERSAM), Instituto de Salud Carlos III, Madrid, Spain*

*5 Department of Clinical Sciences, School of Medicine, University of Barcelona, Barcelona, Spain*

Correspondence to:

Susana Jiménez-Murcia. Department of Psychiatry. Bellvitge University Hospital. E-mail address: [sjimenez@bellvitgehospital.cat](mailto:sjimenez@bellvitgehospital.cat) and Marta Sancho-Navarro. Department of Psychiatry. Bellvitge University Hospital and Hospital de la Santa Creu I Sant Pau. E-mail address: [msanchon@bellvitgehospital.cat](mailto:msanchon@bellvitgehospital.cat)

# Supplementary Data, Figures and Tables

| **Source** | **Studied Addiction**  **Appendix 1.**  **Data items and qualitative synthesis** | **Aim** | **Experimental condition** | **Control condition** | **N** | **Population** | **Mean age (years)** | **Gender (%)** | **Design** |
| --- | --- | --- | --- | --- | --- | --- | --- | --- | --- |
| **Alterman et al. 2014** | SUD | To conduct a pilot investigation of the effectiveness of Mindfulness Meditation (MfM) as an adjunctive treatment for substance abuse patients. | Mindfulness meditation | TAU | 31 | Adults | 36.55 | 45.16% males  54.84% females | Randomized controlled trial |
| **Azizi et al. 2010** | SUD | To investigate the effectiveness of emotional regulation training group therapy (based on DBT and Cognitive Therapy), on improving emotional regulation and distress tolerance skills and relapse prevention in addicts. | Cognitive therapy + Naltrexone  DBT + Naltrexone | Naltrexone | 39 | Males | 26.67 | 100% males | Randomized controlled trial |
| **Bowen and Marlatt, 2009** | Tobacco | To examine effects of a brief mindfulness-based intervention on negative affect, smoking-related urges, and smoking behavior among nicotine-deprived college student smokers. | Mindfulness instruction | No-mindfulness instruction | 123 | College students | 20.33 | 73.2% males  26-8% females | Randomized controlled trial |
| **Bowen et al. 2014** | SUD | To assess the relative efficacy of MBRP, RP and TAU on 12-month SUD outcomes. | MBRP  RP | TAU | 286 | Adults | 38.4 | 71.5% males  28.5% females | Randomized controlled trial |
| **Brewer et al. 2009** | Cocaine and alcohol | To assess MT comparing to CBT in substance use and treatment acceptability and specificity in targeting stress reactivity. | MT base don MBRP | CBT | 36 | Adults | 38.2 | 72% males  28% females | Randomized controlled trial |
| **Brewer et al. 2011** | Tobacco | To assess the efficacy of MTS vs. FFS and correlations between the amount of completed home practice in both treatment arms and smoking outcomes. | MTS | FFS | 88 | Adults | 45.9 | 62.1% males  37-8% females | Randomized controlled trial |
| **Bricker et al. 2014** | Tobacco | To develop the first smartphone app-delivered ACT intervention for smoking cessation (Smart Quit) and to determine (vs. a Quit Guide group control) trial design feasibility, participant receptivity and satisfaction, preliminary cessation outcomes (heavy smoking and low acceptance of craving) and potential impact on acceptance of cravings to smoke. | Smart Quit (ACT) | Quit Guide | 198 | Adults | 41.55 | 48% males  52% females | Randomized controlled trial |
| **Butzer et al. 2017** | SUD | To evaluate the efficacy of a school-based yoga program for reducing substance use risk factors and substance use itself during early adolescence. | Yoga | Physical Education | 211 | Adolescence | 12.64 | 36.8% males  63.2% females | Randomized controlled trial |
| **Carroll H. 2014** | SUD | To assess if mindfulness and/or thought suppression mediates treatment effects on cardiac vagal control in substance abusers. | MBRP  RP | TAU | 34 | Adults | 43.4 | 73% males  27% females | Randomized controlled trial |
| **Chawla et al. 2010** | SUD | To develop a reliable and valid quantitative measure of therapist adherence and competence in delivering MBRP. | MBRP | TAU | 93 | Adults | 40.84 | 64.42% males  35.58% females | Randomized controlled trial |
| **Davis et al. 2013** | Tobacco and alcohol | To test MTS and to see if the mindfulness skills obtained might generalize to decrease alcohol use, in young smokers with regular episodes of binge drinking. | MTS | ILS | 55 | College Students | 21.9 | 70.9% males  29.1% females | Randomized controlled trial |
| **Davis et al. 2014a** | Tobacco | To compare MTS with FFS. | MTS | FFS  Quit Line | 175 | Adults | 44.69 | 52% males  48% females | Randomized controlled trial |
| **Davis et al. 2014b** | Tobacco | To test the treatment acceptability of MTS, to compare MTS with TAU on rates of smoking cessation and to explore possible mediators of effect in the MTS. | MTS | Quit Line | 118 | Adults | 41.65 | 50% males  50% females | Randomized controlled trial |
| **De Dios et al. 2012** | Marijuana | To test the efficacy of a brief intervention using motivational interviewing (MI) + mindfulness meditation (MM) to reduce marijuana use among young adult females. | MI + MM | No-treatment | 34 | Females | 23 | 100% females | Randomized controlled trial |
| **Dixon et al. 2016** | Gambling Disorder | To examine the gambling of college-aged-gamblers (18-2years old) while they actually gambled in an fMRI scanner and to determine the efficacy of ACT at a neurological level. | ACT | No-treatment | 18 | College students (males) | 19.056 | 100% males | Randomized controlled trial |
| **Fishbein et al. 2015** | SUD | SUD To test whether a mindful yoga intervention has a beneficial impact on substance use and its psychophysiological correlates in high-risk adolescents. | Mindful yoga curriculum | TAU (normal class) | 85 | Adolescence | 16.7 | 46% males  54% females | Randomized controlled trial |
| **Garland et al. 2010** | Alcohol | To compare the effects of MORE intervention with a Support Group in adults recruited from a modified therapeutic community. | MORE | Support group | 53 | Adults | 40.3 | 79.2% males  20.8% females | Randomized controlled trial |
| **Garland et al. 2011** | Alcohol | To measure the physiological reactions of substance addicts to external inputs that remind them of the substance is thought to be a predictor of relapse. | MORE | Support group | 53 | Adults | 40.3 | 79.2% males  20.8% females | Randomized controlled trial |
| **Garland et al. 2014** | Opioids | To evaluate the feasibility of developing a clinical trial comparing acute and longer term efficacy of MORE with that of a Support group in reducing chronic pain and prescription opioid misuse. | MORE | Support group | 115 | Adults | 48.35 | 32,18% males  67.82% females | Randomized controlled trial |
| **Garland et al. 2015** | Opioids | To test whether MORE increase the late positive potential (LPP) to natural reward cues in a sample of chronic pain patients at risk for prescription opioid misuse. | MORE | Support group | 29 | Adults | 47.1 | 41.37% males  58.63% females | Randomized controlled trial |
| **Garland et al. 2016** | SUD | To conduce a pragmatic trial to compare MORE as a treatment for substance dependent individuals with trauma histories vs. CBT group and TAU in a modified therapeutic community environment. | MORE  CBT | TAU | 180 | Males | 37.63 | 100% males | Randomized controlled trial |
| **Glasner et al. 2015** | Stimulants | To evaluate the effects of MBRP intervention vs. health education control condition among stimulant dependent adults receiving contingency management. | CM + MBRP | CM + HE | 63 | Adults | 45.3 | 71.4% males  28.6% females | Randomized controlled trial |
| **Glasner et al. 2017** | Stimulants | To compare the incremental efficacy and outcomes of MBRP to an health education control condition for stimulant dependent adults receiving contingency management (CM) and whether results differed between patients with and without affective and anxiety disorders. | CM + MBRP | CM + HE | 63 | Adults | 45.3 | 71.4% males  28.6% females | Randomized controlled trial |
| **Hallgreen et al. 2014** | Alcohol | To explore the feasibility of yoga as part of a treatment program for alcohol dependence. | TAU + Yoga | TAU | 18 | Adults | -- | -- | Randomized controlled trial |
| **Harris et al. 2017** | Alcohol | To investigate whether adding urge surfing as aftercare to a school-based alcohol treatment program improves outcomes in adolescents. | Urge surfing + TAU | TAU | 67 | Adolescence | 16.34 | 78% males  22% females | Randomized controlled trial |
| **Himelstein et al. 2015** | SUD | To study if Mindfulness increases effectiveness of substance abuse treatment in incarcerated youngsters. | Mindfulness + TAU | TAU | 35 | Adolescence (males) | 16.45 | 100% males | Randomized controlled trial |
| **Hsin Hsu et al. 2013** | SUD | To test whether distress tolerance at baseline moderated treatment effects on AOD outcomes over the 4-month follow-up in the context of an initial efficacy trial of MBRP. | MBRP | TAU | 68 | Adults | 40.45 | 63.7% males  36.3% females | Randomized controlled trial |
| **Imani et al. 2015** | Opioids | To assess the effectiveness of Mindfulness-Based Group Therapy (MBGT) compared to TAU in opioid population. | MBGT + TAU | TAU | 30 | Adults | 36.41 | -- | Randomized controlled trial |
| **Kober et al. 2017** | Tobacco | To test whether stress reactivity related to smoking after treatment and at 3-month follow-up; and to compare neural activity during stressful scenarios between treatment groups. | MTS | FFS | 23 | Adults | 48.3 | 69.57% males  30.43% females | Randomized controlled trial |
| **Lee et al. 2011** | SUD | To examine the effectiveness of MBRP on several psychosocial outcome variables among incarcerated illicit drug users who were currently abstinent from illicit drugs. | MBRP | TAU | 24 | Males | 40.70 | 100% males | Randomized controlled trial |
| **Luoma et al. 2012** | SUD | To examine the effects of ACT for shame in SUD. | ACT + TAU | TAU | 133 | Adults | 34 | 54.13% males  45.87% females | Randomized controlled trial |
| **McIntosh et al. 2016** | Gambling disorder | To explore the contribution of a mindfulness-based intervention when delivered with CBT for gambling disorder, and whether the sequencing of these interventions impacted the effectiveness of the treatment. | CBT + Mindfulness  Mindfulness + CBT | TAU | 77 | Adults | 38.48 | 71.4% maes  28.6% females | Randomized controlled trial |
| **Murphy and MacKillop, 2014** | Alcohol | To conduct a laboratory investigation of the effects of a mindfulness strategy on acute craving for alcohol to examine its ability to reduce craving, and to elucidate the domains in which the intervention was primarily exerting its effects. | Mindfulness instruction  Antithetical distraction | Passive control condition | 84 | Adults | 22.43% | 50% males  50% females | Randomized controlled trial |
| **Nakamura et al. 2015** | SUD | To evaluate whether a novel mind-body intervention (Mind-Body Bridging, MBB) is an effective short-term adjuvant intervention for managing SUD and coexisting symptoms in women undergoing residential and outpatient substance use treatment in a community setting. | MBB + TAU | TAU | 38 | Females | 32.55 | 100% females | Randomized controlled trial |
| **Negrei et al. 2015** | SUD | To investigate the possible beneficial effect of introducing mindfulness techniques into a CBT group protocol for diminishing the level of depression and anxiety among a population of clinical participants with addictions. | MCBT | Pharmacotherapy | 60 | Adults | 28.32 | -- | Randomized controlled trial |
| **Price et al. 2012** | SUD | To examine MABT feasibility and to compare with TAU on primary and secondary outcomes. | MABT + TAU | TAU | 46 | Females | 39 | 100% females | Randomized controlled trial |
| **Reza et al. 2014** | Opioids | To examine if MBSR is effective in increasing of Health-Related Quality of Life (HRQOL) in drug-dependent males. | MBSR | No-treatment | 53 | Males | 36.8 | 100% males | Randomized controlled trial |
| **Rogojanski et al. 2011** | Tobacco | To explore whether trait levels of anxiety sensivity and the tendency to respond with symptom-focused anxiety in cigarette cravings moderate smoking-related outcomes after the use of a brief mindfulness or suppression strategy. | Mindfulness strategy | Suppression strategy | 61 | Adults | 40.43 | 59% males  41% females | Randomized controlled trial |
| **Singh et al. 2014** | Tobacco | To extend and test the efficacy of the mindfulness - based intervention in individuals with mild intellectual disabilities. | Mindfulness-based intervention | TAU | 51 | Adults with Mild Intellectual Disability | 33.48 | 80.39% males  19.61% females | Randomized controlled trial |
| **Smallwood et al. 2016** | Opioids | To assess ACT in pain and addiction comorbidity using fMRI to evaluate neurophysiologic alterations across the treatment. | ACT | Health Education | 25 | Adults | 46.8 | 58.3% males  41.7% females | Randomized controlled trial |
| **Stasiewicz et al. 2013** | Alcohol | Alcohol To evaluate the effectiveness of Affect Regulation Training (ART) to address the problems of negative affect drinking and to compare to health education. | CBT + ART | CBT + Health Education | 77 | Adults | 45.7 | 50.65% males  49.35% females | Randomized controlled trial |
| **Tang et al. 2013** | Tobacco | TO evaluate if improved self-control through IBMT reduces craving and smoking. | IBMT | Relaxation | 60 | College students | 21.46 | -- | Randomized controlled trial |
| **Toneatto et al. 2014** | Gambling disorder | To develop M-CBT for problem gamblers and to test its feasibility and initial efficacy. | M-CBT | Wait list | 18 | Adults | 44.1 | 55.55% males  44.45% females | Randomized controlled trial |
| **Valls-Serrano et al. 2016** | SUD | To test the efficacious of Goal Management Training (GMT) + Mindfulness Meditation (MM) group vs. a no-intervention control group in a therapeutic community treatment. | GMT + MM | No-treatment | 36 | Adults | 33.065 | 65.6% males  34.4% females | Randomized controlled trial |
| **Vernig and Orsillo, 2009** | Alcohol | To examine the impact of brief acceptance/mindfulness instructions on emotional responses of alcohol-dependent individuals to pleasant, unpleasant and neutral emotional stimuli. | Acceptance/Mindfulness | TAU  Healthy control | 48 | College students | -- | 44% males  56% females | Randomized controlled trial |
| **Vidrine et al. 2009** | Tobacco | To see the potential associations of mindfulness with nicotine dependence, withdrawal severity and a sense of agency regarding cessation. | MBSR | TAU | 158 | Adults | 43.8 | 55% males  45% females | Randomized controlled trial |
| **Vidrine et al. 2016** | Tobacco | To evaluate the efficacy of MBAT compared to CBT and TAU. | MBAT  CBT | TAU | 412 | Adults | 48.7 | 45.1% males  54.9% females | Randomized controlled trial |
| **Vinci et al. 2014** | Alcohol | To test whether a brief mindfulness meditation can lower levels of negative affect (NA), increase willingness to experience NA, lower urges to drink, and increase time to next alcohol drink in a sample of at-risk college student drinkers. | Mindfulness meditation | Relaxation  Puzzle | 207 | College students | 20.13 | 23.7% males  76-3% females | Randomized controlled trial |
| **Vinci et al. 2016** | Alcohol | To determine whether receiving a brief intervention (mindfulness, relaxation or control) moderate the relationship between baseline level of impulsivity trait and response to the intervention in a sample of at-risk college student drinkers. | Mindfulness meditation | Relaxation  Puzzle | 207 | College students | 20.13 | 23.7% males  76-3% females | Randomized controlled trial |
| **Witkiewitz and Bowen, 2010** | SUD | To examine the relation between measures of depressive symptoms, craving and substance use following MBRP. | MBRP | TAU | 168 | Adults | 40.45 | 63.7% males  36.3% females | Randomized controlled trial |
| **Witkiewitz et al., 2013a** | SUD | To examine the effect of MBRP on levels of craving and changes in craving over time as well as whether changes in acting with awareness, acceptance, and no judgment, mediate the association between MBRP and self-reported changes in craving during and following MBRP. | MBRP | TAU | 168 | Adults | 40.45 | 63.7% males  36.3% females | Randomized controlled trial |
| **Witkiewitz et al., 2013b** | SUD | To evaluate outcomes between non-Hispanic white and racial or ethnic minority women. | MBRP | RP | 105 | Females | 34.1 | 100% females | Randomized controlled trial |
| **Witkiewitz et al., 2014** | SUD | To assess whether MBRP is a feasible and effective intervention at reducing drug use and drug-related consequences in women offenders at a Residential Addiction Treatment Center. | MBRP | RP | 105 | Females | 34.1 | 100% females | Randomized controlled trial |
| **Zemestani and Ottavia, 2016** | SUD | To examine the efficacy of an MBRP vs. TAU in diminishing craving as well as depressive and anxious symptoms among substance abusers in an inpatient treatment setting. | MBRP | TAU | 74 | Adults | 30.1 | 79.7% males  20.3% females | Randomized controlled trial |

ACT: Acceptance and Commitment Therapy; AOD: Alcohol and others drugs; CBT: Cognitive behavioral therapy; DBT: Dialectical Behavior Therapy; FFS: Freedom for Smoking; IBMT: Integrative Body-Mind Training; ILS: Interactive Learning for Smokers; MABT: Mindful Awareness in Body-oriented Therapy; MBAT: Mindfulness-Based Addiction Treatment; MBRP: Mindfulness-Based Relapse Prevention; MBSR: Mindfulness-Based Stress Reduction; M-CBT: Mindfulness-Bases Cognitive Behavioral Therapy; MORE: Mindfulness-Oriented Recovery Enhancement; MTS: Mindfulness Training for Smokers; RP: Relapse Prevention; SUD: Substance Use Disorders; TAU: Treatment as Usual.

**Appendix 2.**

**Data items and qualitative synthesis (continuation)**

| **Source** | **Inclusion criteria** | **Exclusion criteria** | **Outcome measures** | **Technical characteristics of MBIs** | **Main Results** | **Follow-up periods** | **Drop out (n)** | **Limitations** |
| --- | --- | --- | --- | --- | --- | --- | --- | --- |
| **Alterman et al. 2014** | -- | 1. Schizophrenia. 2. Borderline personality disorder. | 1. Life problem areas (ASI). 2. Spirituality (SAS). 3. Personal meaning (LAP-R). 4. Optimism (LOT). 5. Positive mood (PANAS). 6. Positive health (SF-36). 7. Drug use (TLFB). | 8 weeks.  30-45 min group meditation.  7-h workshop on the weekend of week 6. | 1. MfM decreased medical problems over time. 2. Both groups showed significant decreases in alcohol, drug and social problems over time. 3. There was no indication of long-term effects favoring MfM on measures of psychological health (spirituality, personal meaning, optimism, etc.). 4. There were small positive changes at 8 weeks in several areas in MfM group, but they were no longer apparent at 5 months. | 5 months. | 19.36% | 1. Biased sample. 2. Lack of more standard or more intensive substance abuse treatment. 3. Combination of MfM and standard treatment made to obtain with more difficulty a significant effect. |
| **Azizi et al. 2010** | 1. DSM-IV SUD criteria. 2. 20-45 years old. 3. No current psychotherapy. | 1. Psychosis. 2. Organic brain syndrome. 3. Personality disorder. 4. Bipolar disorder. 5. Mental retardation. 6. Major depressive episode. | 1. Opioid treatment. 2. Psychiatric symptoms (GHQ-28). 3. Psychological distress (DTS). 4. Emotion dysregulation (DERS). | 10 sessions.  90-min per session. | 1. Mindfulness and CBT were more effective than naltrexone treatment; and mindfulness was the most effective in increasing distress tolerance and emotion regulation enhancement, in decreasing the amount of drug abuse and psychiatric symptoms. 2. Mindfulness was more effective than CBT increasing distress tolerance and emotion regulation enhancement. | -- | -- | 1. Small sample size. 2. No standard comparison group. 3. Only males. |
| **Bowen and Marlatt, 2009** | 1. ≥ 18 years old. 2. Current cigarette smokers. 3. Interest in cutting down or quitting. | -- | - 1. Smoking and quitting history.   2. Substance dependence (FTND).   3. Affect (PANAS).   4. Smoking urges (QSU).   5. Smoking quantity and frequency. | One experimental session.  90 minutes. | 1. Direct effect of mindfulness instructions on smoking rates over the 7-day follow-up period. 2. No group differences were evident on levels of negative affect or urges during the cue exposure or at the follow-up assessments. 3. Mindfulness practice affect the strength of relation between negative affect and urges, while not necessarily affecting the level of either variable. | 7 days. | -- | 1. Very brief intervention. 2. No smoking cessation intervention. 3. Small smoking reduction in magnitude. 4. No-treatment-seeking population. 5. Self-report measures. 6. Generalizability of the findings. 7. College students and high proportion of males. 8. Assessment procedures. |
| **Bowen et al. 2014** | 1. ≥ 18 years old. 2. English fluency. 3. Medical clearance. 4. Ability to attend treatment sessions. 5. Agreement to random assignment and follow-up assessments. 6. Completion of initial intensive outpatient or inpatient care. | 1. Psychosis. 2. Dementia. 3. Suicidality. 4. Imminent danger to others. 5. Participation in previous MBRP trials. | 1. Substance use. 2. Urianalyses drug and alcohol screenings. | 8 weekly 2-hour sessions.  6-10 participants.  2 therapists. | 1. MBRP and RP (vs. TAU) showed a 54% decreased risk of relapse to drug use and a 59% to heavy drinking, maintaining these effects at the 6-month follow-up and with an advantage of the RP over MBRP on time to first drug use. 2. MBRP (vs. RP) showed a 21% increase in relapse risk to first drug use, but both groups didnt differ significantly on time to the first heavy drinking day. 3. No differences between groups at the 3-month follow-up. 4. At the 12-month follow-up, MBRP (vs. RP) showed significantly fewer drug use days and higher probability of not engaging in heavy drinking. | 3, 6 and 12 months. | 10.53% | 1. Several differences between TAU and treatment groups. 2. Self-report measures. 3. Limited urinalysis data. |
| **Brewer et al. 2009** | 1. English fluency. 2. Meeting DSM-IV criteria for alcohol and/or cocaine abuse or dependence in the past year | 1. < 18 years old. 2. Suicidality or danger to others. 3. Psychosis. 4. Cognitive impairment. 5. Beta-blocker treatment. | 1. SCID. 2. Drug use. 3. Mindfulness (FFMQ). 4. Treatment credibility. 5. Pattern of emotions after stress provocation (DES). | CBT   - 12 weekly 1-hour sessions. - 8 participants.   MT   - 9 weekly 1-hour sessions. - 8 participants.   Both conditions:  1-hour laboratory session with imagery conditions, after 2 weeks of treatment completion. | 1. No differences between CBT and MT in participant retention, treatment satisfaction, or frequency of substance use during the treatment period. 2. MT attenuated psychological and physiological responses to stress provocation (vs. CBT). | Only post-treatment. | 61.89% | 1. Small sample size. 2. Outcome data from the minority of individuals in both conditions who completed treatment. 3. Lack of longer follow-up periods. 4. Heterogeneous population. 5. Single therapists for each condition. 6. No-objective assessment of the amount/quality of treatment. 7. Unequal length (9 sessions vs. 12). |
| **Brewer et al. 2011** | 1. 18-60 years old. 2. Smoking ≥ 10 cigarettes/day. 3. Fewer than 3 months of abstinence in the past year. 4. Interest in quitting smoking. | 1. Psycho-pharmacotherapy. 2. A serious or unstable medical condition in the past 6 months. 3. Meeting DSM-IV criteria for other substance dependence in the past year. | 1. Abstinence. 2. Smoking use (number of cigarettes per day. | 8 sessions: twice weekly 90-min sessions. | 1. MT demonstrated greater reductions in smoking, which were maintained through the 17-week follow-up. 2. Positive relationships between homework completion and substance use outcomes. 3. No correlations between the amount of formal or informal homework in FFS, but strong correlations in MT. 4. No treatment by medication interactions or differences between group correlations between sitting meditation and treatment outcomes. | 6, 12 and 17 weeks. | 29.54% | 1. The study was performed at a single site. 2. Treatment was provided by only 1-2 therapists per condition. 3. Treatment integrity was not formally assessed the exclusion of individuals using psychoactive medication. |
| **Bricker et al. 2014** | 1. ≥ 18 years old. 2. ≥ 5 cigarettes/day for at least past 12 months. 3. Interest in quitting in the next 3days. 4. Interest in learning skills to quit smoking. 5. Agreement to be randomized. 6. Residence in US. 7. Knowledge about downloading a smartphone app from Apples App Store. 8. English fluency. 9. Non other smoking cessation interventions. 10. At least daily access to their own Apple iPhone 4, 4s or 5. | -- | 1. Heaviness smoking index (FTND).  2. Treatment satisfaction.  3. Utilization (number of times they opened their assigned app).  4. Act theory-based acceptance process (AIS).  5. Smoking cessation (TLFB). | Smartphone app-delivered ACT intervention for smoking cessation. | 1. SmartQuit content was more engaging than QuitGuide content. 2. The quit rate differences between groups were more striking among the 2 groups. 3. The treatment would be most beneficial for people who lack skills in accepting their cravings to smoke. 4. The results for heavy smokers are important because this group has very low quit rates and their morbidity and mortality rates are strikingly high. | 2 months. | 32.64% | 1. Small sample size.  2. Short follow-up.  3. Self-reported data. |
| **Butzer et al. 2017** | -- | -- | 1. Mood (BRUMS). 2. Stress (PSS). 3. Impulsivity (UPPS-P); Constructs of Future Time Perspective (FTP) and Present Time Perspective (PTP) from ZTPI). 4. Emotional self-regulation. 5. Substance use willingness, lifetime substance use and frequency. 6. 6. Yoga intervention feasibility (YEQ; CEQ). | 32 sessions.  1- 2 per week over a period of 6 months.  35 min before and after physical education sessions. | 1. Yoga may have beneficial effects on willingness to smoke cigarettes (for males and females) and emotional self-control (for females). 2. 2. No differences between groups pre- to post-intervention, but improvements were observed over an extended period of time. | 1 week, 6 and 12 months. | 2.84% | 1. Loss of consents. 2. It was necessary to do an exam to be admitted. 3. 30.6% of the sample was Asian students. 4. Self-reported outcome measures. 5. Participants were randomized by classroom, instead of individually. 6. Long-treatment. 7. Relatively small magnitude of the significant effects. 8. Small sample size. 9. 9. Transition of patients to other condition complicated the interpretation of the follow-up data. |
| **Carroll H. 2014** | 1. English fluency. 2. 18-70 years old. 3. Medical clearance. 4. Completed intensive treatment. 5. Agreement to random assignment. | 1. Psychosis. 2. Suicidality. | 1. V02max. 2. Anxiety (STAI). 3. Present state craving levels. 4. Mindfulness (FFMQ). 5. Though suppression (WBSI). 6. Addiction severity (ASI). 7. Hemodynamics. 8. Laboratory stressor (PASAT). | 8 weekly sessions. | 1. MBRP was significantly positively related to mindfulness, whereas RP was significantly inversely related to mindfulness. 2. MBRP was associated with higher levels of some factors of mindfulness and RP with higher levels of though suppression. 3. Those who practiced mindfulness tended to produce psychophysiological responses against stress (self-regulation and self-soothing behaviors instead of abusing substances. | 2 months | 3.4% | 1. Small sample size. 2. Lack of the necessary power to detect modest significant effects. 3. Almost all self-reported measures. |
| **Chawla et al. 2010** | -- | -- | 1. Adherence and competence to MBRP (MBRP-AC). 2. Therapeutic alliance. 3. Mindfulness (FFMQ). 4. Rater selection and training. 5. Rater guidelines. | 8 weekly 2-hours sessions.  2 therapists.  6-10 participants. | 1. High interrater reliability for all treatment adherence and competence subscales of the MBRP-AC. 2. High level of internal consistency for therapist style and competence ratings. 3. The facilitation of exercises and practices in MBRP increase mindful . 4. Lack of 5. between therapist competence and participant mindfulness/ratings of working alliance. 6. Relatively high levels of adherence for an initial feasibility and efficacy trial. 7. 100% adherence to the key concepts of MBRP and high rating in the competence. 8. Sufficient reliability and validity of the MBRP-AC to assess therapist treatment delivery. | -- | -- | 1. Non assessment of the treatment discriminabillity. 2. Only assessed prescribed therapist behaviors and not those that are proscribed by the MBRP treatment manual. 3. The use of nave raters, who are not experienced providers of MBRP. |
| **Davis et al. 2013** | 1. 18-20 years old. 2. Smoking ≥ 10 cigarettes/day. 3. ≥ 5 drinking binges per month. | 1. ≥ 4 drinks on ≥ 6 nights per week. 2. Psychosis. | 1. Substance use (TLFB). 2. Intervention completion (Quit Day Retreat). 3. Practice compliance. 4. Nicotine dependence (FTND). 5. Smoking motives (WISDM). 6. Mindfulness. 7. Distress tolerance (DTS). 8. Stress (PSS). 9. Course acceptability. | 6 weekly 2-hour sessions and a 7-hour Quit Day Retreat on the weekend between sessions 4 and 5. | - 1. Smoking abstinence: - Point prevalent smoking abstinence was higher in MTS (vs. controls), but the difference was not significant. - MTS showed significantly greater number of days abstinent in the first two weeks vs. ILS.   1. Alcohol use: controls significantly increased alcohol consumption over the course of their intervention (vs. MTS).   2. Alcohol use and smoking relapse: Post-quit alcohol use was significantly associated with smoking relapse in every measure obtained.   3. High attrition rates.   4. Acceptability: Class attendance was reasonable for completers; minutes meditated and minutes walked were also reasonably high for completers. | 2 weeks. | 54.55% | 1. High attrition. 2. Small sample size. 3. Outcomes were assessed only at 2-weeks post-quit. 4. The magnitude and pattern of treatment group differences at long-term follow-up are unknown. |
| **Davis et al. 2014a** | 1. Living in low socieconomic areas. 2. ≥ 18 years old. 3. Smoking ≥ 5 cigarettes/day. 4. No others tobacco products. 5. High motivation to quit. 6. ≤ 4 alcohol drinks on more than 4 days per week. | -- | - 1. Class attendance.   2. Attrition.   3. Practice compliance.   4. Smoking abstinence.   5. Urge intensity.   6. Mindfulness acquisition.   7. Psychological outcomes. | 7 weekly 2 and half-hours sessions and 6 and half-hour Quit Day Retreat. | 1. MT produced similar smoking abstinence rates post-treatment vs. controls, but at 24-weeks post-quit attempt, numeric abstinence rates diverged favoring the MT. 2. MT was associated with decreased urges, increased mindfulness, and decreased stress and experiential avoidance. | 4 and 24 weeks after quit day. | Post-treatment: 29.62%.  At 4-weeks follow-up: 35.83%.  At 24 weeks follow-up: 57.53%. | 1. The participants were not blinded to their treatment conditions. 2. High attrition rates. 3. The possibility with any lengthy intervention there will be change in motivation over time. |
| **Davis et al. 2014b** | 1. ≥ 18 years old. 2. Smoking ≥ 5 cigarettes/day. 3. No others tobacco products. 4. High motivation to quit. 5. Willingness to attend 10 treatment sessions over a 2-month period | 1. ≥ 4 drinks on ≥ 4 nights per week. 2. PHQ-9 ≥ 9 (Suicidality and major depression). | Primary outcomes:   1. Biologically confirmed 7-day point prevalence abstinence.   Secondary outcomes:   1. Meditation calendar. 2. Smoking history, smoking abstinence (FTND) and smoking motives (WISDM). 3. MTS Course Evaluation. 4. Emotion regulation (DERS). 5. Attentional control (ACS). 6. Mindfulness (FFMQ). | 7 weekly 90-min sessions and 4 weekday sessions. | - 1. Primary outcomes: - Point prevalence abstinence at 4 and 24 weeks post quit attempt did not reach significance in the intent-to-treat analysis, but did reach significance in analysis of treatment initiators.   1. Potential change processes:      - MTS showed significant improvement on self-report measures of emotion regulation, attentional control, and mindfulness over the treatment period.      - Post-treatment scores on these measures were significantly correlated with meditation time and smoking abstinence. | 4 and 24 weeks. | Post-treatment: 39.79%.  At 4-weeks follow-up: 46.93%.  At 24 weeks follow-up: 71.94%. | - 1. Lack of a time/intensity matched control.   2. No-blinding.   3. High attrition rates. |
| **De Dios et al. 2012** | 1. Smoking marijuana at least 3 times in the past month. 2. Female. 3. 18-29 years old. 4. Living within 20 miles of Providence. 5. Planning to remain in the geographic area for the next 3 months. 6. English speaker. 7. Endorsing a desire to quit or reduce the marijuana use. 8. Using marijuana as a strategy for emotion regulation. | 1. Comorbidity with other psychiatric disorders that can interfere with treatment (schizophrenia, untreated bipolar disorder, posttraumatic stress disorder). 2. Using alcohol or other substances at high levels (7 alcohol drinks/week in the past month). 3. Using other drugs in the past month. | 1. Substance use (TLFB). 2. Generalized anxiety disorder (PDSQ). | 2 individual 45-min sessions with 2 weeks between both. | 1. MI+MM intervention decreased marijuana use by 6 to 8 days per month (vs. control group). 2. This decrement was sustained at the 3-month follow-up. 3. Only 2 women achieved full marijuana abstinence at the 3-month follow-up. | 1, 2 and 3 months. | 1 month: 20-7%  2 months: 23.5%  3 months: 26.5% | 1. Lack of and attention-matched comparison condition. 2. Lack of determination about active components of the intervention. 3. Self-reported measures. 4. Small sample size and only young women. 5. Low levels of treatment and follow-up attrition. |
| **Dixon et al. 2016** | 1. SOGS ≥ 3. 2. 18-20 years old. | -- | Primary measures:   1. Brain activation patterns (BOLD signal). 2. Subjective ratings of slot machine outcomes during the fMRI task.   Secondary measures:   1. AAQ-II. 2. VLQ. 3. MAAS. | 8 weekly 1-h sessions. | - 1. ACT group (vs. control) showed greater brain activation patterns for winning spins when compared to the initial scanning sessions.   2. All gamblers at the initial fMRI scan showed decreased activation in the dopaminergic activation centers, including the amygdale, cuneus, and ventral striatum.   3. The neurological change observed in the treatment group resembled similar brain activation patterns in regions as non-pathological gamblers.   4. At post-test ACT group showed an increased activation in the middle frontal gyrus and interior parietal lobule.   5. Following treatment, ACT group was more likely to report higher engagement in psychological flexibility and mindfulness-related behaviors.   6. In both groups, lower MAAS scores overall, reporting the control group the largest decreases in MAAS scores over time.   7. Over time, ratings for winning and losing outcomes differed significantly in the treatment group (vs. control). | -- | -- | 1. Treatment dose. 2. Manualized nature of treatment delivery. 3. Subjective ratings of closeness to a win. 4. A very restricted age range of non-treatment seeing problem gamblers. |
| **Fishbein et al. 2015** | 1. ≥ 18 years old. 2. Students in high-recovery school. | -- | - 1. Health and fitness status (PARQ; ACASI).   2. Dysregulation (ADI).   3. Stress (RSQ).   4. Mood (BMS).   5. Mindfulness (FFMQ).   6. Drug use (DUSI-R).   7. Behavior (BASC-2).   8. 8. Physiological activity. | 20 sessions.  50 min per session.  3 days each week for 7 weeks. | 1. In the control group (vs. experimental group), the alcohol use decreased in the past month post-treatment, whereas it was increasing over time. 2. Marginally significant results in change over time for 4 outcomes: alcohol use, social skills and two measures of stress reactivity as reflected in SC responses. 3. The control group had less social skills and more SC vs. the experimental group (more social skills and less SC). 4. No differences between groups on mindfulness autoperceived levels, emotion regulation, involuntary engagement coping (disengagement rumination and emotional numbing). | -- | 18.2% | 1. A pilot study. 2. Small sample size. 3. Attrition rate sign. 4. Irregular attendance of participants to yoga sessions. 5. Control students didn’t receive an active treatment because this school did not offer regular PE classes. 6. Biased teacher reports. 7. No sufficient average dosage to affect some regulatory processes. |
| **Garland et al. 2010** | 1. ≥ 18 years old. 2. Meeting DSM-IV alcohol dependence criteria. 3. Residence in the therapeutic community for ≥ 18 months. | -- | - 1. Intervention credibility.   2. Mindfulness (FFMQ).   3. Psychosocial factors related to alcohol dependence (BSI, PACS, IRISA, PSS-10, and WBSI).   4. Psychophysiological cue-reactivity.   5. Alcohol attentional bias. | 10 sessions. | 1. MORE reduces stress and alcohol thought suppression to a significantly greater extent (vs. ASG). 2. MORE decreases alcohol bias towards brief visual alcohol cues and increases HRV recovery from alcohol cues following stress induction. 3. MORE appears to be a feasible intervention to prevent stress-precipitated alcohol relapse, based on the high perceived treatment credibility and program adherence, and the absence of significant differences in attrition from an ASG. 4. Contrary to the hypotheses, MORE did not significantly increase self-reported mindfulness, nor did it result in significant decrements in craving. 5. Both interventions led to statistically significant reductions in psychiatric symptoms. | -- | 30.18% | 1. Small sample size. 2. Limited generalization of results. 3. Self-report measures. 4. 4. Lack of follow-up data. |
| **Garland et al. 2011** | 1. Alcohol-dependent adults in long-term residential treatment. 2. ≥ 18 years old. 3. Meeting DSM-IV alcohol dependence criteria at the time of admission. 4. Residential treatment for ≥ 18 months. | -- | - 1. Alcohol use (AUDIT).   2. Psychiatric symptoms.   3. Alcohol attentional bias.   4. 4. Affect-modulated cue reactivity protocol. | 10 weeks. | 1. Both metrics significantly and independently predicted odds of relapse up to 6 month post-treatment. 2. Relapsers had significantly higher HFHRV reactivity compared to those who did not relapse. 3. 3. There was no statistically significant alcohol attention bias or AB differences between groups in bivariate analyses (except for the mean, higher for relapsers). | 2 and 4 weeks. | 13.11% | 1. Lack of the independent systematic biochemical assessment of abstinence from alcohol to verify reports of relapse from the treatment facility. 2. Limited statistical power. 3. 3. No real control group. |
| **Garland et al. 2014** | 1. Recurrent pain stemming from chronic benign (non-cancer related) pain conditions. 2. Treatment with opioid analgesics daily or nearly every day for at least the past 90 days. | 1. Suicidality. 2. Psychosis. | 1. Pain Severity and Pain interference (BPI). 2. Craving. 3. Self-reported opioid misuse (COMM). 4. Non-reactivity (FFMQ). 5. Reinterpretation of pain sensations (CSQ). | 8 weekly 2-h sessions.  8-12 participants. | 1. MORE significantly reduced symptoms associated with chronic pain and prescription opioid misuse and self-reported sympathetic stress arousal, maintaining these reductions for 3 months follow-up. 2. MORE significant increased in non-reactivity and reinterpretation of pain sensations. 3. MORE reduced craving for opioid. 4. Both interventions decreased self-reported opioid misuse. | 3 months. | 40.04% | 1. Inability to quantify differences in opioid dose. 2. Lack of quantitative tracking of non-adherence and assessment of the relationship between treatment fidelity and outcome. 3. Use of different therapists for interventions. 4. High attrition rates. 5. Lack of measurement of a circumscribed subset of these manifold treatment targets. |
| **Garland et al. 2015** | 1. Recurrent pain stemming from chronic benign (non-cancer related) pain conditions. 2. Treatment with opioid analgesics daily or nearly every day for at least the past 90 days. | 1. Suicidality. 2. Psychosis. | 1. Electrocortical activity. 2. Affective images (IAPS). 3. Event-related brain potentials. 4. Opioid craving (COMM). | 8 weekly 2-h sessions. | - 1. MORE modulated natural reward processing in opioid (mis)using chronic pain patients by enhancing the LPP at the stage of attentional allocation.   2. After completing 8 weeks of treatment, MORE group exhibited a heightened LPP response to natural reward stimuli relative to neutral stimuli (vs. SG).   3. Increases in electrocortical activity during reward processing predicted enhanced positive affective responses to the photographs and reductions in opioid craving from pre- to post-treatment.   4. Repeated practice of conscious, top-down modulation of attentional allocation onto the positive features of a stimulus context can amplify ERPs during motivated attention to natural reward stimuli. | -- | -- | 1. Lack of a quantitative measure of opioid dose. 2. Multimodal intervention. 3. Small and heterogeneous sample. 4. LPP results might have been attenuated because the athletic photos from the IAPS tend to elicit weaker LPPs. |
| **Garland et al. 2016** | 1. ≥ 18 years old. 2. Current DSM-IV SUD and psychiatric disorder diagnosis. 3. Homelessness prior to entering into the therapeutic community. | 1. Psychosis. 2. Substance withdrawal. | 1. Trauma history. 2. Craving (PACS). 3. Post-traumatic stress symptoms (PCL-C). 4. Psychiatric distress (BSI). 5. Dispositional mindfulness (FFMQ). 6. Positive and negative affect (PANAS). 7. Readiness to change (URICA). | 10 weekly 2-h sessions. | - 1. MORE was associated with modest yet statistically significant improvements in craving, post-traumatic stress symptoms, and positive and negative affect from pre- to post- treatment.   2. A significant indirect effect of MORE on craving and post-traumatic stress through increased dispositional mindfulness.   3. 3. CBT was not found to significantly improve craving or post-traumatic stress symptoms. | -- | 28.89% | 1. The duration of the therapeutic benefits is unknown due to not follow-up. 2. Low-dose provision of coping skill training to TAU may have obscured differences between the CBT and TAU groups. 3. Psychometrics of the trauma history measure have not been evaluated. 4. 4. Lack of biochemical measures of abstinence. |
| **Glasner et al. 2015** | 1. ≥ 18 years old. 2. Current DSM-IV diagnosis of stimulant dependence. 3. English fluency. 4. Being physically able to sit for 3minutes. | 1. Medical and/or psychiatric impairment. 2. Medical detoxification. 3. Homeless. | 1. Urine drugs screen (stimulant use). 2. Addiction Severity Index (ASI). 3. Psychiatric severity (BDI). | 12 weeks.  4 weeks CM + 8 weeks MBRP or HE. | 1. MBRP group greater declines in negative affect and psychiatric severity. 2. Among those with Major Depression and GAD, stimulant use declined significantly in response to MBRP (vs. control condition). 3. Effectivity in reducing stimulant use among those with concomitant major depression and GAD. | 1 month. | -- | -- |
| **Glasner et al. 2017** | 1. ≥ 18 years old. 2. Current DSM-IV diagnosis of stimulant dependence. 3. English fluency. 4. Being physically able to sit for 30 minutes. | 1. Medical and/or psychiatric impairment. 2. Medical detoxification. 3. Homeless | 1. Stimulant use (urine drug screens weekly). 2. Negative affect (BDI; BAI). 3. Psychiatric severity (ASI). | 8 weekly 75-min sessions. | - 1. MBRP improved depressive and anxiety symptoms and reduced overall severity of psychiatric impairment among stimulant dependent adults.   2. MBRP is particularly helpful to stimulant users with MDD and GAD in reducing stimulant use.   3. Group differences in measures of emotion regulation and acquisition of mindfulness skills at mid- and end-of-treatment. | 1 month | 57.14% | 1. Small sample size. 2. Diagnostic instrument. 3. No-generalizabillity to other mood and anxiety disorders. 4. Drop-out. 5. Ceiling effect of CM on stimulant use outcomes. |
| **Hallgreen et al. 2014** | 1. DSM-IV alcohol dependence criteria. 2. ≥ 18 years old. 3. Men and women. | 1. Major social problem (social services). 2. Severe withdrawal symptoms or a serious mental illness (psychosis, bipolar disorder). | 1. Alcohol use. 2. Affective symptoms (HAD). 3. Health-related functioning and quality of life (SDS). 4. Stress (PSS and saliva cortisol). | 10 weekly 90-min group yoga sessions. | 1. At six-month follow-up, positive changes in alcohol use, affective symptoms and quality of life in both groups. 2. The yoga intervention reported a larger reduction in alcohol use but without significance (the primary outcome measure). 3. When the yoga intervention was combined with standard treatment, the urge to drink reduced noticeably. 4. Stress reduction and mood enhancement appear to be the primary mechanism responsible for these changes. | 6 months | -- | 1. Small sample size. |
| **Harris et al. 2017** | Have been participant in a school-based intervention program. | Non having used alcohol within the previous 90 days prior to starting the school-based intervention. | Alcohol use. | 4 weekly 1-h sessions. | Experimental group showed significant reductions in alcohol quantity and frequency during the aftercare period. | 4 weeks. | 19.40% | 1. Self-reported measures. 2. Poor rate at which diary cards were returned in subsequent sessions. 3. Lack of categorization between students whose goals were abstinence vs. reduction of use. |
| **Himelstein et al. 2015** | -- | -- | 1. Mindfulness (MAAS). 2. Locus of control (PLOCS). 3. Decision making skills (DMS). 4. Self-esteem (SES). 5. Lifestyle. 6. Behavioral regulation. | 8-12 weeks, 90 min per session. | 1. Mindfulness + TAU showed a significant greater increase of self-esteem (vs. TAU). 2. Significant changes on behavior among groups over time:  - An improvement in Mindfulness + TAU. - A decrease in TAU.  1. A significant increase in decision making skills and self-esteem among all participants. | -- | 38.63% | 1. Small sample size. 2. Possible confounded data due to the context of the detention camp. 3. Lack of generalization of the results. 4. 4. The direct difference method to examine significant differences between groups. |
| **Hsin Hsu et al. 2013** | -- | 1. Psychosis. 2. Dementia. 3. Suicidality. 4. Significant risk of withdrawal from AOD. 5. Need for more intensive treatment. 6. Non completing inpatient or intensive outpatient treatment. 7. Non attending MBRP groups due to scheduling conflicts. 8. < 8 weeks until completion of aftercare. | 1. Drug use (TLFB). 2. Distress tolerance (DTS). 3. Midnfulness (FFMQ). | 8 weekly 2-hours group sessions. | 1. DTS was positively associated with all mindfulness subscales. 2. Support for the use of the DTS in a clinical sample with drug disorders. 3. Individuals with lower distress tolerance who received MBRP reported greater reductions In AOD use frequency over time (vs. TAU). 4. The changes were not maintained at the 4-month follow-up. 5. Drug use days rebounded at the 4-month follow-up to be similar among individuals with higher distress tolerance, regardless of conditions. | Post-treatment, 2 and 4 months. | -- | 1. TLFB is a self-report retrospective method. 2. The brevity of the follow-up period. 3. Attrition. 4. Difficulty to compare homework in the MBRP (e.g., meditation practice) and TAU (e. g., working through 12 steps) conditions given the differences in content and format. |
| **Imani et al. 2015** | 1. DSM-IV-TR opioid dependence criteria. 2. 18-4years old. 3. ≥ 8 years of completed education. 4. Completion of 2 weeks of medical treatment with opioid agonist medication. 5. Informed written consent for participation in the study. | 1. Psychosis. 2. Dementia. 3. Suicidality. 4. Organic brain disorders. 5. Diagnosis of other drug dependence (except nicotine). | 1. Clinical condition (ASI). 2. Mindfulness (FFMQ). | 8 weekly 120-min sessions. | 1. More significant improvement in substance use in MBGT (vs. TAU). 2. There were differences between groups in all subscales of mindfulness. 3. Effectiveness of mindfulness techniques in treatment of opioid dependents. 4. Efficacy of MBGT for decreasing substance use. | -- | 6.67% | 1. No recorded sessions. 2. Small sample size. 3. Only one consultant. |
| **Kober et al. 2016** | 1. English fluency. 2. 18-60 years old. 3. Smoking ≥ 10 cigarettes/day. 4. < 3 months of abstinence in the prior year. 5. High motivation to quit. 6. No claustrophobia, colorblindness, history of severe head trauma with loss of consciousness, neurological disorders, or any MRI-contraindicated conditions (e.g., metallic implants). | 1. Non being able to read and understand the entire consent form. 2. Psycho pharmacotherapy. 3. A serious or unstable medical condition in the prior 6 months. 4. Meeting DSM-IV criteria for other substance dependence in the past year. | 1. Average number of cigarettes per day. 2. Self-reported abstinence. 3. Pre-treatment stress reactivity (PSS). | 2 weekly group sessions over 4 weeks.  fMRI stress task following the treatment. | 1. Neural reactivity in regions including amygdale and insula related to smoking outcomes after treatment and at 3 month follow-up. 2. MT showed lower stress reactivity in these regions. 3. Reduction in tress reactivity as a mechanism of MT treatment related change. | 3 months. | -- | 1. Small sample size. 2. Lower signal-to-noise ratio from scanner. 3. No available self-report ratings for the full sample due to technical difficulties during data acquisition. 4. Individuals participated in the fMRI session after completing smoking cessation treatment, and changes from pre-to post-treatment were impossible to assess. 5. No collection of the information about substance use except cigarettes and alcohol. |
| **Lee et al. 2011** | 1. 1-year sentences due to possession or sale of illicit drugs. 2. Use illicit drugs in the past. 3. Abstinence for 6 months or more. | 1. Psychosis. 2. Delirium. 3. Illiteracy. | 1. Drug use (DUDIT-E). 2. Drug avoidance. 3. Depression (BDI-II). | 10 group sessions.  1.5h per session. | 1. Baseline measures: MBRP (vs. TAU) showed less positive attitudes towards substance use and less frequently used drugs before incarceration. 2. MBRP (vs. TAU) showed significantly higher negative expectancies of use. 3. MBRP decreased depressive mood. 4. No differences post-treatment regard to positive expectancies or self-efficacy. 5. Main effect of Group by Time on negative outcome expectancies between MBRP and TAU. | Post-treatment | 0 | 1. Small randomized clinical trial. 2. Lack of longer-term follow-up assessments. 3. Only male participants. 4. Depression was measured weekly in the MBRP group only. |
| **Luoma et al. 2012** | -- | -- | Primary outcomes:   1. Internalized Shame Scale (ISS). 2. Treatment Services Review (TSR). 3. Substance use (TLFB).   Secondary outcomes:   1. General Health Questionnaire – 12 (GHQ). 2. Quality of Life Scale (QLS). 3. Multidimensional Scale of Perceived Social Support (MSPSS). | 28 days residential program.  2h session in 3 days during 1 single week (6h in total). | 1. Reductions in shame during active treatment predicted higher levels of substance use at follow-up. 2. The more gradual reductions in shame found in the ACT group protected against the pattern seen in TAU. 3. ACT intervention led to higher levels of outpatient treatment attendance during follow-up, which in turn were functionally related to lower levels of substance use. 4. ACT group showed a pattern of continuous treatment gains, especially on psychosocial measures. | 4 months. | Post-treatment: 15.03%  4 months: 46-15% | 1. Missing data. 2. Severe substance abusing populations can be difficult to track through follow-up. 3. TAU in a residential program. 4. The measure of shame was limited to the ISS. 5. The comparability of this sample to others is unclear. 6. TLFB data were not collected pre-treatment. 7. Lack of blinding. |
| **McIntosh et al. 2016** | -- | 1. Psycho pharmacotherapy for depression and/or anxiety and non willingness to maintain the dosage throughout the study. 2. Concurrent psychotherapy. 3. Severe substance misuse or dependence criteria. 4. Severe mood disorder, psychosis and/or suicidality. | 1. Gambling behavior (DSM; SOGS). 2. Psychological distress (DASS-21). 3. Mindfulness (FFMQ). 4. Rumination and thought suppression (RRQ and WBSI). 5. Quality of life (SF-12). 6. Treatment satisfaction and homework and effectiveness of treatment (CSQ). | 8 weekly sessions.  60 minutes per session, except the first (90 minutes). | 1. Mindfulness and TAU interventions were effective at reducing PG behavior and associated distress, quality of life, certain mindfulness facets, thought suppression and rumination. 2. The combination of psycho-education, mindfulness intervention and CBT may be a useful supplement to traditional CBT treatments. 3. PG behavior and secondary outcome measures became significantly lower at the 6-month follow-up vs. post-treatment in the MF-first group. | 3 and 6 months. | -- | 1. Early randomized trial. 2. Small sample size. 3. The ethics requirement to provide assessment and psycho-education. 4. Brief Manualized CBT and mindfulness treatment (4 sessions each other). 5. Loss of follow-up data. |
| **Murphy and MacKillop, 2014** | 1. At-risk heavy drinker (>14 or 7 drinks per week for men and women respectively. 2. AUDIT ≥ 8. 3. 21-29 years old. | -- | 1. Craving (PACS). 2. Alcohol use and misuse (DDQ; AUDIT). 3. Urge distress (DRSEQ). 4. Mood. 5. Psychophysiological arousal (heart rate). 6. Mindfulness (FFMQ). | Mindfulness instruction set. | 1. Mindfulness strategy was not significantly better at reducing desire to drink or urge distress. 2. Mindfulness and acceptance-based strategies to reduce acute craving were not superior to a control condition. 3. Independently of group assignment, individuals reported greater confidence in their ability to resist urge in a variety of high-risk situations. 4. At 1 week follow-up, participants reported lower levels of craving and reduced alcohol consumption. | 1 week. | -- | 1. Lack of alcohol self-administration component. 2. Different modality to collect data in the laboratory session (self-reported questionnaire) vs. during follow-up (telephone assessment). 3. The coping strategy manipulations were acute in nature and did not involve extensive practice. 4. Lack of generalization of results. |
| **Nakamura et al. 2015** | 1. Current substance abuse treatment program. 2. English fluently. | 1. Active psychosis. 2. Suicidality. | Primary measures:   1. Craving (PACS). 2. Severity and incidence (SIP-AD).   Secondary measures:   1. Subjective distress. 2. Depression. 3. Sleep problems. 4. Mindfulness (FFMQ). 5. Self-compassion (SCS). 6. Well-being (WBI). 7. Expectation for treatment benefit. | 2 weekly 2-hours sessions over 10 weeks.  20 sessions in total. | 1. MBB + TAU decreased the primary outcome measure of drug and alcohol cravings. 2. MBB + TAU decreased the impact of past trauma (especially with respect to avoidance) and disturbed sleep (vs. TAU). 3. MBB + TAU increased mindfulness, self-compassion and well-being (vs. TAU). 4. While MBB was effective in decreasing self-reported depressive symptoms, there was no statistically significant difference between the two groups, as both interventions led to a decrease in depression symptoms. | -- | 18.42% | 1. Logistical difficulties inherent in a community-based setting. 2. No follow-up assessment. |
| **Negrei et al. 2015** | -- | -- | 1. Depression (BDI).  2. Anxiety (HAD). | 12 sessions, 2 per month.  4 hours per session. | 1. CBT group had lower scores on BDI and HAD vs. medication group. 2. The combination of mindfulness and CBT techniques was efficient in diminishing the level of depression and anxiety. | -- | -- | -- |
| **Price et al. 2012** | 1. Enrollment in the outpatient program. 2. Willingness to sign a release to contact. 3. Willingness to forego further nonstudy massage or bodywork during the first 3 months of study involvement. 4. Ability to commit to a regular scheduled time to attend MABT sessions. 5. Willingness to accept random assignment to study treatment conditions. | 1. Current domestic violence. 2. Pregnancy beyond 2 months. | Primary outcomes:   1. Substance use (TLFB). 2. Reasons for relapse (RDQ).   Secondary outcomes:   1. Psychological distress. 2. Physical distress. 3. Stress and coping. 4. MABT process measures. 5. Body awareness practice at 6-month follow-up. | 8 weekly 90-min sessions. | 1. MABT (vs. TAU) was superior at the 3-month follow-up in substance use. 2. MABT reduced craving and use in response to social pressure. 3. MABT had significant improvements in eating disorder symptoms, depression, anxiety, and frequency of physical symptoms. 4. MABT reduced dissociation, perceived stress, and emotion regulation difficulties of control and limited strategies relative to women in TAU. | 3, 6 and 9 months. | 43.47% | 1. No control for time and attention. 2. Small sample size. 3. DERS findings may not be valid without the use of the entire scale. 4. The sample had a higher socioeconomic status and functional ability. 5. Only women. |
| **Reza et al. 2014** | 1. Drug-dependent males in addiction treatment center. 2. Opium or heroin dependent. 3. Methadone maintenance treatment during the study. | -- | 1. Quality of life (HRQOL). | 8 weekly 90-min group sessions. | 1. MBSR was a useful method for enhancing the HRQOL in drug-dependent males. 2. Results showed an association of cultivating a more mindful way of being with less emotional distress and a more positive state of mind. 3. Increased awareness of thoughts and emotions, acceptance, and compassion appear to promote optimal HRQOL. | -- | 7.55% | 1. Small sample size. 2. Limited generalization of the results. |
| **Rogojanski et al. 2011** | 1. Smoking ≥ 10 cigarettes/day over the past month. 2. ≥ 18 years old. 3. Interest in cutting down or quitting smoking, or have tried to quit smoking in the past. | -- | 1. Anxiety. 2. Self-efficacy. 3. Smoking use and dependence (FTND). | -- | 1. Higher state symptom-focused anxiety after the intervention was associated with greater self-efficacy 1 week later in the suppression condition, and lower self-efficacy within the same period of time in the mindfulness condition. 2. State symptom-focused anxiety immediately after the cue induction procedure was a significant predictor of self-efficacy at the follow-up. 3. Dispositional anxiety sensitivity did not impact on outcomes. | 7 days. | 20% | 1. Small homogeneous no-treatment-seeking sample. 2. Symptom-focused anxiety has generally not been conceptualized as a state variable, and the measure of state symptom focused anxiety adapted for the study requires validation. 3. Drop-out. |
| **Singh et al. 2014** | 1. Adult with mild intellectual disability. 2. Ability to give own informed consent. 3. Willingness to enter a smoking cessation study. 4. Approval by their primary care physician to engage in this smoking cessation program. 5. Willingness to engage in mindfulness training or continue with their current treatment. 6. Willingness to work with their Support coordinator to collect smoking data. | Axis I psychiatric diagnosis (DSM-IV). | 1. Smoking use (number of cigarettes per week). 2. Drop-out. | -- | 1. No differences between groups in drop-out rates. 2. A statistically significant number of treatment completers stopped smoking in the experimental group (vs. control group). 3. During the 1-year follow-up, the mean number of cigarettes smoked was smaller in the experimental group (vs. control group). | 1 year. | 21.57% | 1. Reported observational data on the number of cigarettes smoked. 2. Relatively modest sample sizes. 3. Lack of mindfulness measure prior to and following intervention. |
| **Smallwood et al. 2016** | 1. Patients with chronic low back pain persisting ≥ 12 months. 2. Meeting DSM-IV criteria for opioid dependence. 3. Opioid replacement therapy. | -- | - 1. Brain MRI.   2. Psychological flexibility.   3. Mindfulness (MAAS).   4. Disability due to back pain.   5. 5. Pain intensity, pain interference and opioid craving. | 8 sessions over 4 weeks. | 1. There were neurophysiologic effects following the ACT in patients with CLBP and OA comorbidity. 2. Reduced activation in regions commonly involved in pain processing indicates that ACT may decrease the brain’s responsiveness to painful stimuli in patients with comorbid CLBP and OA. 3. ACT group:  - Had decreased connectivity at rest post-treatment compared with pre-treatment and with the HEC group. - Had more differing connections and stronger differences in those connections in the pain network.  1. ACT may target DMN function at rest more than connectivity between pain-related regions. | -- | -- | 1. Small sample size. 2. The most conservative corrected statistical thresholds could not be used. 3. Difficulty for generalization. |
| **Stasiewicz et al. 2013** | 1. Seeking outpatient alcohol treatment services. 2. Current DSM-IV alcohol dependence diagnosis. 3. Negative affect drinking profile. 4. Living within community distance of the program site. | 1. Acute psychosis. 2. Use of medications (disulfiram, naltrexone) that may modify alcohol use. 3. Made changes in past 3 months in dose or type of prescription medication that affects mood. 4. Any drug use diagnosis other than for nicotine and cannabis. 5. Legally mandated to attend treatment. | 1. Abstinence. 2. Substance use (drinks per day). 3. Heaviness of drinking. 4. Behavioral variables (coping strategies, drinking situations). 5. Affective variables (negative affect, anxiety and mood symptoms, affect regulation). 6. Cognitive variables (self-efficacy). 7. Attendance, satisfaction and working alliance throughout treatment. | 12 weekly 90-min sessions. | 1. ART showed significantly greater increases in percent days abstinent from baseline to end of treatment that began to slow and slightly decline during follow-up (vs. HLS). 2. ART significantly reduced drinks per drinking day at the 3-month follow-up (vs. HLS). 3. ART did not significantly differ at any of the time points for percent heavy drinking days. 4. A significant difference between groups at end-of-treatment for the affect regulation, but not at baseline (increase in ART group). 5. ART showed significant increases in describing and significant decreases in negative affect (vs. HLS). | 3 and 6 months. | Post-treatment:  22.125%  3 months:  31.205%  6 months:  36,365% | 1. Limited results on measures of emotion regulation. 2. Lack of identification of the more active components of ART. 3. Homogeneity/heterogeneity of the sample. |
| **Tang et al. 2013** | College students that want to learn meditation/ relaxation to reduce stress and improve cognitive performance. | Goal of quitting smoking. | 1. Smoking use (FTND, exhaled CO level). 2. Imaging data acquisition and analysis. | 10 consecutive sessions over 2 weeks, with 30 min of practice (5 hours in total). | 1. Significant smoking reduction in the IBMT group (vs. RT). 2. IMBT group (vs. RT) showed significantly:  - An increase of the activity at ACC/medial PFC and inferior frontal gyrus/ventrolateral PFC (vs. RT). - A decrease activity at PCC/precuneus, cerebellum, and other regions after training (vs. RT). - A decrease of craving. | 2 and 4 weeks. | 3.33% | 1. Lack of adequate control conditions. 2. Failure to randomize participants. 3. Lack of assessment of biological markers of change. |
| **Toneatto et al. 2014** | 1. Pathological gambling diagnosis. 2. Willingness to practice mindfulness meditation. | 1. Substance abuse/dependence (except nicotine). 2. Concurrent attendance at any treatment in which gambling was addressed (including Anonymous Gamblers). | 1. Gambling severity. 2. Gambling urges. 3. Emotional distress (BSI). 4. Trait mindfulness (KIMS). | 5 sessions: 3 weekly sessions and 2 every other week.  90-min per session. | 1. At baseline, no significant differences between groups in gambling severity, psychiatric symptoms, gambling urges or trait mindfulness. 2. At post-treatment, M-CBT reported significantly fewer DSM gambling symptoms, maintaining the therapeutic gains at the 3-month follow-up. 3. Diagnostic criteria for pathological gambling were also significantly reduced at the 3-month follow-up. 4. Mindfulness practice showed significantly fewer DSM symptoms, gambling urges and psychiatric symptoms. 5. The number of minutes of mindfulness practice was significantly correlated with a reduction in psychiatric symptoms. | 3 months. | 22.22% | 1. Control group (wait list). 2. Small sample size. 3. The absence of mindfulness data post-treatment. 4. Lack of corroboration of the dependent measures. 5. 5. Absence of direct measures of gambling behavior severity. |
| **Valls-Serrano et al. 2016** | 1. DSM-IV criteria for SUD. 2. Abstinence period ≥ 15 days. 3. Absence of psychiatric comorbidity on Axis I and Axis II (less nicotine dependence). 4. Absence of a history of head injury and neurological, infectious, systemic or any other nervous system. | 1. Medical impairment that compromised their safety. 2. Medical detoxification from any substances. 3. Psychiatric impairment that warranted hospitalization or primary treatment. 4. Homeless. | 1. Basic executive functions. 2. Ecological measures of planning and multitasking. | 8 weekly 2-h sessions. | GMT + MM vs control group:   1. Achieved goals in daily activities. 2. Improved working memory, reflection-impulsivity/decision-making and performance. 3. Reduced stress levels. 4. Increased in planning time. | -- | 13.89% | 1. Small sample size. 2. Lack of follow-up periods. 3. Possible differences between groups. |
| **Vernig and Orsillo, 2009** | 1. Dependent group: alcohol dependence criteria. 2. Comparison group: drinking ≤ 2 drinks per day on average and no DSM-IV-TR alcohol dependence criteria. | 1. ≤ 18 years old or unable to sign a legal document. 2. Significant visual impairment. 3. Difficulties reading English. | 1. Emotional reactions (SAM). 2. Alcohol use (AUDIT). 3. Substance use disorders (DAST-10). 4. Acceptance/mindfulness (manipulation and strategies). | A single session. | 1. No differences between groups in emotional reactions. 2. Absence of self-reported heightened emotional reactivity within the alcohol dependent groups. 3. No impact of the acceptance/mindfulness instructions on self-reported emotional responding. 4. Mindfulness group scored significantly higher on the strategies. 5. Alcohol dependence severity was predicted by intensity of psycho-physiological response to unpleasant slides. 6. More severe alcohol dependence was significantly associated with a tendency to label the unpleasant (but not the neutral or pleasant) slides as more unpleasant. | -- | -- | 1. Small sample size. 2. No generalization due to type of sample (college students). |
| **Vidrine et al. 2009** | 1. ≥ 5 cigarettes per day for the past year. 2. Motivated to quit smoking within the next 30 days. 3. English fluencly. 4. A viable home address and phone number. | 1. Lack of motivation to quit within 30 days. 2. Regular use of tobacco products other than cigarettes. 3. Use of nicotine replacement and smoking < 5 cigarettes per day. 4. Contraindication of nicotine patch. 5. Enrollment in another cessation program. 6. Pregnancy or lactation. 7. Psychotropic medication use. 8. Another household member enrolled in the study. | 1. Smoking motives, heaviness and withdrawal. 2. Self-efficacy. 3. Affective information processing. 4. Mindfulness (KIMS and MAAS). | 8 weekly group sessions. | 1. Age and years of smoking were the only demographic smoking variables significantly associated with the degree of mindfulness. 2. Greater degree of mindfulness was associated with lesser nicotine dependence and withdrawal severity. 3. Degree of mindfulness was overall positively associated with self-efficacy ratings regarding one’s ability to abstain from smoking in high-risk situations. | -- | 0% | 1. Cross-sectional study. 2. Because withdrawal severity was assessed prior to cessation, participants were likely not in physiological withdrawal at the time of the assessment. 3. Selection bias. |
| **Vidrine et al. 2016** | 1. ≥ 18 years old. 2. ≥ 5 cigarettes per day for the past year. 3. Motivated to quit smoking within the next 30 days. 4. A viable home address and phone number. 5. English fluency. 6. An expired air CO level ≥ 8 ppm. 7. Provided colateral contact information. | 1. Contraindication for nicotine patches use. 2. Regular use of tobacco products other than cigarettes. 3. Use of bupropion or nicotine replacement products other than the study patches. 4. Pregnancy or lactation. 5. Another household member enrolled in the study. 6. Active substance dependence. 7. Current psychiatric disorder or use of psychotropic medication. 8. Participation in a smoking cessation treatment program in the previous 90 days. | 1. Smoking dependence. 2. Mindfulness (MAAS and KIMS). 3. Mindfulness technique practice during treatment. 4. Smoking abstinence. 5. Lapse recovery. | 8 2-hours group sessions. | 1. No significant overall differences in abstinence rates across the three treatments. 2. MBAT showed benefits over and above CBT and TAU in promoting recovery from a lapse among participants who were not abstinent at the end of treatment. | 4 and 26 weeks. | 58.3% | 1. Specialization on the part of therapists. 2. High level of engagement on the part of patients. 3. Treatment diffusion bias. 4. Lack of data to establish fidelity by therapists. 5. Low rates of compliance with formal meditative practices. 6. Lack of information about use of the nicotine patch. 7. Definition of “lapse recovery”. 8. Attrition. |
| **Vinci et al. 2014** | 1. AUDIT ≥ 6. 2. Endorsement of drinking primarily for Enhancement and Coping Motives on the DMQ-R. | 1. AUDIT ≤ 6. 2. No endorsement of drinking primarily for Enhancement and Coping Motives on the DMQ-R. | 1. Positive affect and Negative affect. 2. Urge to drink (AUDIT; DMQ-R). 3. Mindfulness (FFMQ; TMS). | 5-h per day, 5 days per week. | 1. Mindfulness was effective at increasing state mindfulness (vs. relaxation and control groups). 2. Physical relaxation was effective at significantly increasing level of relaxation for the relaxation and mindfulness group (vs. control group). 3. After NA induction, more NA in TAU. 4. Relaxation group showed lower levels of PA following the relaxation intervention (vs. Mindfulness and TAU). 5. NA increased for all groups and Urge for the mindfulness and relaxation, following the NA induction. 6. No significant differences between groups in willingness to watch images and time to next drink. | -- | -- | 1. Lack of generalization to other populations. 2. Small sample size. 3. Brief interventions. 4. Drinking urges and drinking behavior were not assessed. 5. Lack of follow-up assessment of affect or urges. |
| **Vinci et al. 2016** | 1. AUDIT ≥ 6. 2. Elevated score on at least one of two subscales of the DMQ-R. | -- | 1. Alcohol use (AUDIT; DMQ-R). 2. Mindfulness (FFMQ; TMS). 3. Impulsivity (UPPS-P). 4. Affect (PANAS). 5. Urge to drink. | 10-min task (mindfulness, relaxation or puzzle). | 1. In mindfulness intervention, higher levels of Sensation Seeking, Negative and Positive Urgency facets were associated with higher levels of negative affect and urge, and lower levels of positive affect. 2. In mindfulness intervention, the facet of (lack of) Perseverance decreased and was associated with increased Positive Affect. | -- | -- | 1. Lack of the generalization of the sample. 2. Weak strength of correlations. 3. Lack of follow-up periods. 4. Self-reported measure to gather level of impulsivity. 5. Lack of mindfulness assessment. |
| **Witkiewitz and Bowen, 2010** | 1. English fluency. 2. Completed intensive outpatient or inpatient treatment in the previous 2 weeks. | 1. Suicidality. 2. Active psychosis. 3. Inability to participate due to scheduling conflicts. 4. < 8 weeks until completion of aftercare. 5. No-completed inpatient or intensive outpatient treatment. | 1. Substance use (TLFB). 2. Craving (PACS). 3. Depression (BDI-II). 4. Mindfulness integrity (MBRP-AC). | 8 weekly 2-h sessions.  6-10 participants.  2 therapists. | 1. Positive relations between post-intervention depressive symptoms, craving at the 2-month follow-up, and days of alcohol or other drug use over the 4-month follow-up. 2. Craving significantly mediated the relation depressive symptoms – alcohol and drug use days following intervention in TAU (vs. MBRP). 3. Depressive symptoms moderated the relation between intervention assignment and craving 🡪 no association in MBRP vs. a strong association in TAU. | 2 and 4 months | Post-treatment: 20.83%.  2 months: 27.38%.  4 months: 26.78%. | 1. Small sample sixe. 2. Brevity of the post-intervention follow-up. 3. A brief mindfulness intervention may be not enough to maintain the long-term effects. 4. Missing data at follow-up assessment points. 5. The rates of abstinence might not generalize to non-mandated treatment populations. 6. Self-reported measures. 7. Training of the therapists. 8. The different number of treatment hours per week between both groups. 9. No blind to intervention group assignment. 10. 10. No comparison with other empirically supported active treatments. |
| **Witkiewitz et al., 2013a** | 1. 18-70 years old. 2. English fluency. 3. Completed intensive outpatient or inpatient treatment in the previous 2 weeks. 4. Medical clearance. | 1. Psychosis. 2. Dementia. 3. Suicidality. 4. Withdrawal risk. 5. Need for more intensive treatment. 6. No completed inpatient or intensive outpatient treatment. | 1. Substance craving. 2. Mindfulness (FFMQ). 3. Acceptance (AAQ). | Weekly 2-h sessions.  6-10 participants.  2 therapists. | 1. MBRP showed lower craving scores during and following treatment. 2. MBRP scored higher on AAQ and FFMQ indicating a tendency toward greater acceptance and less judgment. 3. Non-Hispanic white was associated with higher levels of craving and a greater decrease in craving over time. 4. 4. The combination of processes (acceptance, awareness and nonjudgment) is necessary to predict changes in craving. | 2 and 4 months. | -- | 1. Self-reported measures. 2. Brevity of the follow-up. 3. Lack of a no-treatment or wait list control group. 4. Missing data. 5. 5. Noteworthy differences between TAU and MBRP groups, with respect to therapist training, group composition and group content. |
| **Witkiewitz et al., 2013b** | 1. English proficiency. 2. Willingness to be randomized. 3. Ability to provide consent. | -- | - 1. Substance use (TLFB).   2. Addiction Severity (ASI). | 2 weekly 50-min sessions over 8 weeks. | 1. Racial or ethnic minority participants MBRP reported no drug use days and significantly lower addiction severity at 15-week follow-up (vs. RP). 2. Lower rates of medical problems among racial and ethnic minority participants in MBRP. 3. 3. Significantly better follow-up rates in racial and ethnic minority vs. non-Hispanic white participants assigned to MBRP (85.7% vs. 52.6%). | 15 weeks. | Post-intervention: 32.10%.  Follow-up: 48.6%. | 1. Small sample size. 2. High attrition rates. 3. Lack of race and ethnicity data. 4. Treating minority participants as one group vs. assessing racial and ethnic groups. 5. No look at outcomes by the intersection of race, ethnicity and gender simultaneously. 6. All drugs of abuse were combined into a single outcome measure, preventing assessment of differential effects of treatment for different substances. 7. Some women remained in a controlled environmental during follow-up. |
| **Witkiewitz et al., 2014** | 1. Residency at the treatment center. 2. English proficiency. 3. Willingness to be randomized. 4. Sufficient self-reported cognitive ability to understand and provide consent. | -- | Primary outcomes:   1. Substance use (TLFB). 2. Consequences of substance use (SIP-M).   Secondary outcomes:   1. Social and family problems, legal issues and psyquiatric symptoms (ASI). | 2 weekly 50-min sessions over 8 weeks. | Primary outcomes:   1. MBRP had 96% fewer days of drug use vs. RP. 2. Drug use consequences were 39% lower in MBRP, without statistical significance.   Secondary outcomes:   1. MBRP had significantly lower scores on the legal status and medical status subscales of the ASI. 2. No significant differences on the family/social subscale or the psychiatric status subscale. 3. 3. No significant differences on ASI total score. | 15 weeks. | Post-intervention: 32.10%.  Follow-up: 48.6%. | 1. Small sample size. 2. High attrition rates. 3. Small-to-medium effect size differences in treatment outcomes for MBRP vs. RP, without statistical significance. 4. Missing data. 5. Design limitations as non-recording of sessions. |
| **Zemestani and Ottavia, 2016** | 1. DSM-IV-TR criteria for substance dependence for the past year. 2. A minimum of 2 weeks in the inpatient or outpatient treatment center and detoxification. 3. BDI-II > 20. 4. To be able to speak and read Persian. | 1. Psychotic disorder. 2. Suicidality. 3. Significant withdrawal risk. 4. Need for more intensive treatment or didn’t complete inpatient or outpatient treatment. | 1. Depression (BDI-II). 2. Anxiety (BAI). 3. Alcohol craving (PACS). | 8 weekly 2-h sessions.  12-13 participants. | Significantly higher pre-treatment to post-treatment improvements in depression, anxiety and craving symptoms in MBRP (vs. TAU). | 2 months | 14.86% | 1. Arab sample. 2. Self-reported outcome measures. 3. Short follow-up. 4. Some patients didn’t meet DSM-IV major depressive disorder criteria. 5. 5. No other empirically supported active treatment as a control group. |

AAQ: Acceptance and Action Questionnaire (Bond et al., 2011); ACASI: Audio Computer-Assisted Self- Interview; ACC: Anterior Cerebral Cortex; ACS: Attentional control Scale (Derryberry & Reed, 2002); ACT: Acceptance and Commitment Therapy; ADI: Abbreviated Dysregulation Inventory (Mezzich et al., 1997); AOD: Alcohol and others drugs; ART: Affect Regulation Training; ASG: Addiction Support Group; ASI: Addiction Severity Index; AUDIT: Alcohol Use Disorders Identification Test (Babor et al., 1992); BAI: Beck Anxiety Inventory; BASC-2: Behavior Assessment Scale for Children (Reynolds and Kamphaus, 2004); BDI: Beck Depression Inventory; BMS: Brunel Mood Scale (Terry et al., 2003); BOLD: Blood Oxygen Level Dependent; BPI: Brief Pain Inventory; BRUMS: Brunel University Mood Scale); BSI: Brief Symptoms Inventory (Derogatis, 1993); CBT: Cognitive behavioral therapy; CLBP: Chronic Low-Back Pain; CEQ: Control Evaluation Questionnaire; COMM: Current Opioid Misuse Measure (Butler et al., 2007); CSQ: Coping Strategies Questionnaire (Rosenstiel & Keefe, 1983); DASS-21: Depression, Anxiety and Stress Scales 21-item version; DAST-10: Drug Abuse Screening Test (Gavin, Ross & Skinner, 1989); DBT: Dialectical Behavior Therapy; DERS: Difficulties in Emotion Regulation Scale (Gratz & Roemer, 2004); DES: Dissociation Experiences Scale; DMN: Default Mode Network; DMQ-R: Drinking Motives Questionnaire-Revised; DRSEQ: Drinking Refusal Self-Efficacy Questionnaire; DSM: Diagnostic and Statistics Manual; DTS: Distress Tolerance Scale (Simons & Gaher, 2005); DUDIT-E: Drug Use Identification Disorders Test (Berman et al., 2007); DUSI-R: Drug Use Screening Inventory – Revised (Tarter and Kirisci, 2001); FFMQ: Five Facet Mindfulness Questionnaire (Baer et al., 2006); FFS: Freedom for Smoking; fMRI: functional Mangenitc Resonance Imaging; FTND: Fagerstrom Test of Nicotine Dependence (Heatherton et al., 1991); GAD: Generalized Anxiety Disorder; GHQ-28: General Heatlh Questionnaire (Goldberg, 1972); GMT + MM: Goal Management Training + Mindfulness Meditation; HAD: Hospital Anxiety and Depression Scale; HE: Health Education; HFHRV: High-Frequency Heart Rate Variability; HLS: Healthy and lifestyle; HRQOL: Health-Related Quality of Life; IAPS: International Affective Picture System; IBMT: Integrative Body-Mind Training; ILS: Interactive Learning for Smokers; IRISA: Impaired Alcohol Response Inhibition Scale (Guardia et al., 2007); KIMS: Kentucky Inventory of Mindfulness; LAP-R: Reker’s Life Attitude Profile-Revised (Reker, 1992); LOT: Life Orientation Test (Scheier and Carver, 1985); MAAS: Mindfulness Attention Awareness Scale; MABT: Mindful Awareness in Body-oriented Therapy; MBAT: Mindfulness-Based Addiction Treatment; MBB: Mind-Body Bridging; MBGT: Mindfulness-Based Group Therapy; MBRP: Mindfulness-Based Relapse Prevention; MBRP-AC: Mindfulness-Based Relapse Prevention – Adherence and Competence Scale; MBSR: Mindfulness-Based Stress Reduction; M-CBT: Mindfulness-Bases Cognitive Behavioral Therapy; MDD: Major Depression Disorder; MfM: Mindfulness Meditation; MI: Motivational Interview; MORE: Mindfulness-Oriented Recovery Enhancement; MT: Mindfulness Training; MTS: Mindfulness Training for Smokers; NA: Negative Affect; OA: Opioid Addiction; PA: Positive Affect; PACS: Penn Alcohol Craving Scale (Flannery, Volpicelli & Pettinati, 1999); PANAS: Positive and Negative Schedule (Watson, Clark & Tellegen, 1988); PARQ: Physical Activity Readiness Questionnaire; PASAT: Paced Auditory Serial Addition Test; PCC: Posterior Cerebral Cortex; PCL-C: 17-item PTSD Checklist-Civilian Version (Weathers et al., 1993); PDSQ: Psychiatric Diagnostic Screening Questionnaire (Zimmerman & Mattia, 2001); PG: Pathological Gambling; PHQ-9: Patient Health Questionnaire – 9; PLOCS: Parental Locus of Control Scale; PSS-10: Perceived Stress Scale (Cohen, Kamarck & Mermelstein, 1983); QSU: Questionnaire of Smoking Urges (Cox, Tiffany & Christen, 2001); RMDQ: Roland Morris Disability Questionnaire (Roland and Morris, 1983); RP: Relapse Prevention; RRQ: Rumination Reflection Questionnaire; RSQ: Response to Stress Questionnaire (Connor-Smith et al, 2000); RT: Relaxation Training; SADD: Short Alcohol Dependence Data Questionnaire; SAM: Self Assessment Manikin (Bradly & Lang, 1994); SAS: Spirituality Assessment Scale (Howden, 1992); SCS: Self-Compassion Scale (Neff, 2003); SF-36: Health Survey Questionnaire (Ware et al., 1995); SDS: Severity of Dependence Scale; SG: Support Group; SIP-AD: Short Inventory of Problems – Alcohol and Drugs (Blanchard et al., 2003); SIP- M: Short Inventory of Problems (Bennett et al., 2009); SOGS: South Oaks Gambling Screen (Lesieur & Blume, 1987); STAI: State-Trait Anxiety Inventory; SUD: Substance Use Disorders; TAU: Treatment as Usual; TLFB: Timeline Follow-Back (Brown et al., 1980); TMS: Toronto Mindfulness Scale (Lau et al., 2006); UPPS-P: Urgency, Perseverance, Premeditation and Sensation Seeking Impulsive Behavior Scale (Cyders & Smith, 2007; Whiteside & Lynam, 2001); VLQ: Valued Living Questionnaire (Wilson et al.,. 2011); WBI: World Health Organization Well-Being Index (Bech et al., 2003); WBSI: White Bear Suppression Inventory; WISDM: Wisconsin Inventory of Smoking Dependence Motives (Smith et al., 2010); YEQ: Yoga Evaluation Questionnaire; ZTPI: Zimbardo Time Perspective Inventory.
